# Nonlinear control of a fully actuated robotic hand using high-order sliding mode and feedback linearization controllers

**DOI:** 10.1371/journal.pone.0333512

**Published:** 2025-10-17

**Authors:** Asra Sarwat, Wenjie Lu, Maryam Iqbal

**Affiliations:** 1 Department of Mechanical Engineering and Automation, Harbin Institute of Technology, Shenzhen, University Town, China; 2 Department of Electrical Engineering, Bahria University, H-11, Islamabad, Pakistan; Southwest University of Science and Technology, CHINA

## Abstract

The increasing adoption of prosthetic devices in medical applications introduces complex and variable load conditions, particularly due to the diverse nature of user disabilities. To address the resulting control challenges, this paper proposes a novel High-Order Fully Actuated Sliding Mode Controller (HOFA-SMC) implemented to enhance robustness under system uncertainties and non-linearities. The proposed controller incorporates a proportional-integral (PI) framework to structure the high-order terms and effectively mitigate the chattering commonly associated with sliding mode control. Stability of both the HOFA-SMC and a feedback Linearization controller (FLC) is established using Lyapunov theory. A detailed simulation study is conducted on a full hand model, comprising four 4-degree-of-freedom (DOF) fingers and a 3-DOF thumb, implemented in Python. The controllers are evaluated across three test scenarios: flexion, extension, and ball grasping. Results indicate that HOFA-SMC achieves rapid trajectory convergence (within 0.2 s) and robust performance under varying uncertainty conditions. A comparative analysis further confirms the superiority of HOFA-SMC over traditional SMC and FLC approaches in trajectory tracking and control stability.

## Nomenclature

*SMC*
: Sliding Mode Control
*HOFA-SMC*
: High-Order Fully Actuated Sliding Mode Control
*FLC*
: Feedback Linearization Control
*ADL*
: Activities of Daily Living
*MAS*
: Multi-Agent Systems
*CDNN*
: Coupled Discontinuous Neural Networks
*LMI*
: Linear Matrix Inequality
*PMSM*
: Permanent Magnet Synchronous Motors
*AVS*
: Active Vertical Suspension
*RBF-SMC*
: Radial Basis Function Sliding Mode Control
*MIMO*
: Multi-Input Multi-Output
*NMST-SMC*
: Novel Modified Super-Twisting Sliding Mode Control
*FSR*
: Force-Sensitive Resistor
*MP*
: Metacarpo Phalangeal
*FES*
: Functional Electrical Stimulation
*AFGFTSMC*
: Adaptive Fuzzy Global Fast Terminal Sliding Mode Control
*SOSM*
: Second-Order Sliding Mode
*GAN*
: Generative Adversarial Networks
*FL*
: Feedback Linearization
*PIO*
: Proportional Integral Observer
*FL-GPC*
: Feedback Linearization-Based Generalized Predictive Control
*PIP*
: Proximal Interphalangeal Joint
*IP*
: Interphalangeal Joint
*FJRs*
: Flexible Joint Robots
*IBC*
: Inversion-Based Control
*CSTR*
: Continuous Stirred-Tank Reactor
*MSE*
: Mean Square Error
*ABC*
: Artificial Bee Colony
*MGABC*
: Modified Genetic Artificial Bee Colony
*ST-FSMC*
: Self Tuning Fuzzy Sliding Mode Controller
*PID*
: Proportional Integral Derivative
*LQR*
: Linear Quadratic Regulator
*DOF*
: Degrees of Freedom
*GOAL*
: Generalized Object Grabbing and Alignment Learning
*GNet*
: Grabbing Network
*GRAB*
: Grabbing and Retrieval of Articulated Bodies
*RGMCs*
: Robotic Grasping and Manipulation Competitions

## 1 Introduction

The increasing prevalence of limb disabilities, due to congenital conditions, accidents, or hazardous work environments, has led to a growing demand for advanced prosthetic devices capable of restoring hand functionality and enabling individuals to perform activities of daily living (ADLs) [2,6]. A person with partial or complete loss of hand function may struggle with essential tasks such as grasping, holding, or manipulating objects. The development of robotic prosthetics, particularly those mimicking human hand biomechanics, has therefore become a critical focus of medical and engineering research [4].

Biomechanics, the study of mechanical principles applied to biological systems, plays a foundational role in prosthetic design and control. It facilitates a deeper understanding of the forces, torques, and kinematic requirements needed to replicate human motion [1]. Despite significant advances in mechanical design, control of anthropomorphic hands remains a complex problem due to the nonlinear, uncertain, and multi-degree-of-freedom nature of the human hand. Control systems for prosthetic hands must address several challenges, including variability in load conditions, time-varying dynamics, and external disturbances. Linear controllers are often inadequate for such applications. As real-world systems are inherently nonlinear, nonlinear robust control methods—particularly Sliding Mode Control (SMC)—have been extensively studied for prosthetic and robotic systems [3]. SMC offers advantages such as robustness to parameter variations and external disturbances; however, traditional SMC suffers from chattering and may not generalize well to high-precision tasks.

Recent studies have introduced various modifications to enhance the performance of SMCs. These include integral SMC for systems with time delays and uncertainties [7], adaptive SMC for neural network synchronisation [8], and fractional-order SMCs for electric motor control [9]. In high-speed applications, disturbance observers have been integrated with SMC to suppress vibrations in vertical suspension systems [10]. For robotic finger coordination, radial basis function (RBF)-based sliding mode controllers (SMCs) with hyperbolic tangent functions have improved chattering suppression and trajectory tracking [11]. Moreover, fuzzy logic and neural networks have been employed to estimate system uncertainties and reduce reliance on full state measurements [12]. Similarly, in wind energy systems, modified super-twisting algorithms have demonstrated improved robustness and dynamic response [13].

Despite these advancements, the problem of high-precision nonlinear control in prosthetic hands, particularly under uncertain and unbalanced load conditions, remains only partially addressed. Most approaches either compromise on tracking performance or fail to eliminate chattering without increasing complexity.

In [14], the authors have proposed a distributed fixed-time synchronisation controller using neuro-adaptive non-singular terminal sliding mode control for higher-order multi-agent nonlinear systems. The controller utilises radial basis function neural networks to manage unknown dynamics and alleviate uncertainties, facilitating fast sliding-mode enforcement while minimising chattering. In [15], the authors discuss the importance of developing haptic grippers and hands for performing accurate object manipulation in applications such as minimally invasive surgery, prosthetics, and industrial automation. The authors propose a two-finger robotic hand equipped with force-sensitive resistor (FSR) sensors and servomotor current averaging for enhanced gripping accuracy and speed, as well as reduced damage or slippage. Integration of force-sensitive resistor (FSR) sensors with servomotor current averaging.

In [16], the authors propose a robotic gripper that features force control, tactile sensing, three-dimensional perception, and an autonomous manipulation framework encompassing detection, segmentation, force-controlled manipulation, and symbolic replanning. The design exhibits robustness, diversity, and utility in research, teaching, and automation, as confirmed by tasks such as gear assembly and sensor-based stacking.

In [17], the authors introduced that Multi-fingered robotic hand-in-hand manipulation is typically hindered by the limited set of finger postures that can be achieved due to the traditional design of stiff, link-based fingers. Such fingers, often constructed from stiff links of constant length and revolute joints, restrict the flexibility and dexterity of the robotic hand, limiting its ability to perform complex tasks such as component assembly and precision handling. To overcome this restriction, continuum robots have been proposed as a means of enabling robotic fingers to achieve a flexible, continuous range of motion. Continuum robots, which bend, extend, and retract, are free from the constraints of rigid links and can take on numerous postures, making robotic hands more dexterous. The continuum robots, achieved through the continuous curvature approach, provide increased flexibility and reach, as demonstrated by simulations and grasping experiments. These designs surpass conventional two-link robotic fingers by greatly expanding the space that can be grasped and by enabling more diverse contact with objects. As a result, the integration of continuum robots into multi-fingered hands has the potential to substantially enhance the dexterity and utility of robot systems across applications.

In [18], the authors have proposed a technique in which functional electrical stimulation (FES) is used to activate the forearm muscles, and feedback control principles have enhanced the accuracy of motion in the metacarpophalangeal (MP) joint of the index finger. A proxy-based super-twisting algorithm (PSTA) is presented for accurate MP joint servo control. This approach mitigates windup during FES saturation and ensures robust control precision by integrating a second-order super-twisting algorithm with first-order sliding mode control. The implicit Euler method reduces numerical chattering in digital systems. The efficacy of the proposed strategy is substantiated via experimental validation using an Arduino and human participants. Successful sliding mode control (SMC) systems must possess essential qualities, including stability, the elimination of chattering, speed, and a short convergence time. In [19], the authors introduce a task space control strategy for robot manipulators with dynamic and kinematic uncertainty, specifically the adaptive fuzzy global fast terminal sliding mode control (AFGFTSMC). The system employs a global fast terminal SMC with a tunable sliding surface to improve convergence time. It also includes a seven-rule fuzzy approximator for intelligent coefficient tuning, which further boosts convergence. Robustness is enhanced, and the five-rule adaptive fuzzy approximator prevents chattering. Finite-time global asymptotic stability is proved using theoretical analysis, and simulations on a 2-link robot manipulator confirm the effectiveness and robustness of the controller.

In [20], the authors have proposed a novel approach to modeling, simulation, and analysis of two-finger biomechanics, with an application example illustrating its use in activities of daily living. Two nonlinear control methods, Sliding Mode Control (SMC) and Feedback Linearization Control (FLC), are employed to produce accurate and stable finger movement in a two-degree-of-freedom model. These controls successfully synchronize flexion and extension movements, taking into account physiological constraints and eliminating nonlinearities such as changes in loads, velocity variations, and damping forces. MATLAB/Simulink simulation validates the approach. The outcome illustrates the viability and robustness of the suggested methodology.

In [21], the authors have proposed a work that utilizes proprioceptor data and neural inputs to develop physiologically suitable optimal controllers that mimic the decision-making of the central nervous system (CNS). A biomechanical framework in the human palm reference frame is simulated to study finger movement coordination. It utilizes a 21-DOF biomechanical model that considers sensory noise, environmental disturbances, and physiological dynamics. The tracking of the fingertip trajectory reduces the model order to 18 states using an $H_{\infty }$ control paradigm. Flexion movement of a robotic finger is analyzed under disturbances, with feedback forces at the joints allowing stabilization at a flexion angle of 1 rad/s within 2 seconds. The reduced-order model faithfully follows the reference trajectory. With applications in kinesiology, ergonomics, assistive technology, and prosthetics, this work sheds light on the coordination of hand movement.

The nonminimum phase characteristic of a boost converter in continuous conduction mode presents challenges for the voltage regulation system. To handle these complications, the work in [22] utilizes accurate feedback linearization. An adaptive second-order sliding mode (SOSM) controller is proposed and designed using the Lyapunov framework to regulate voltage in the presence of disturbances. By adaptively modifying the control gain, the controller reduces overestimation and minimizes chattering, a phenomenon prevalent in conventional SOSM approaches. Compared to other methods, finite disturbances are needed instead of their derivatives. The suggested controller ensures finite-time stability, enhances transient response, and improves robustness. Comparative hardware experiments confirm its superiority and effectiveness.

A novel method for generating a feedback linearization controller (FLC) for uncertain systems using generative adversarial networks (GANs) is discussed in [23] the authors have proved that reference tracking is enhanced by optimizing the FLC using a minimax scheme, where an adversarial strategy is employed to estimate system uncertainty based on ground truth samples from an accessible integral model. Theoretical guarantees ensure convergence and stability, facilitating robust recovery of the FLC. The method employs an augmented adversarial loss function and a strictly convex generator structure to prevent mode collapse during GAN training. The suggested method has been verified and proven superior and effective through comprehensive experiments and simulations.

The authors in [24] have presented an Event-triggered dynamic feedback linearization control strategy for nonlinear systems, aiming to mitigate internal disturbances caused by sampling errors and ensure the stability of the internal dynamics. Stability is assessed through a polytopic differential inclusion model, and a trigger function is used to remove disruptions. The strategy enables co-designing controllers through a multi-objective optimization problem, where the region of attraction is maximized and transmissions are minimized. Numerical examples provide detailed demonstrations of the effectiveness.

A robust control approach utilizing feedback linearization (FL) is proposed in [25] for flexible joint robot systems with uncertainty. To address problems in real systems, a proportional-integral observer (PIO) is proposed for estimating the state vector and uncertainty, encompassing modeling errors, parameter variations, and external disturbances. The proposed FL-based PIO controller eliminates the need for the whole state vector and enhances performance in uncertainty. Closed-loop stability is ensured, and simulations confirm the effectiveness of the PIO in state estimation and tracking. Experimental confirmation of the approach’s effectiveness is presented using the Quanser flexible joint module.

Regarding non-linear control strategies, authors in [26] developed a two-degree-of-freedom dynamic model for manipulator trajectory tracking control to overcome the difficulties associated with applying generalized predictive control (GPC) to nonlinear systems. A feedback linearization-based generalized predictive control (FL-GPC) is proposed. The method combines iterative nonlinear variable estimation and predictive linear system control. Simulation results for the manipulator’s static and dynamic trajectory tracking problems demonstrate the FL-GPC method’s validity for high-precision control.

A robust feedback linearization technique that controls robot manipulators is proposed in [27] using a first-order Taylor series expansion to linearize the dynamics. A redesigned PD control algorithm with Taylor-series compensation guarantees robust reference tracking. The method demonstrates that bounded nonlinear states exhibiting exponential convergence to a bounded set are derived from stable linearized dynamics. The methodology is corroborated by trials with a 4-DOF exoskeleton and a 1-DOF robot.

A feedback linearization controller with fuzzy adaptive sliding modes is used for trajectory tracking of a flexible robot manipulator in [28]. Feedback linearization is used to linearize nonlinear dynamics and apply sliding mode control for stability. Gradient descent and chain derivative techniques adapt the controller settings, while the Takagi–Sugeno–Kang fuzzy system alters the gains. Fuzzy rules are refined using a multi-objective particle swarm optimization method that reduces control effort and state error. The simulation results illustrate the efficacy of the suggested strategy compared to other approaches.

Each finger of a robotic hand has many joints that replicate human anatomy. The metacarpophalangeal joint (MCP) is located between the hand and the proximal part of the finger, facilitating flexion and extension. The Proximal Interphalangeal Joint (PIP) is located between the first and second phalanges of the finger, facilitating flexion and extension at the middle segment. The Distal Interphalangeal Joint (DIP) is between the second and third phalanges of the finger, providing mobility at the fingertip. The thumb contains specialized joints, starting with the Carpometacarpal Joint (CMC), which offers wide-ranging mobility, including opposition. The thumb’s metacarpophalangeal joint (MCP) provides flexion and extension, and the Interphalangeal Joint (IP) controls mobility at the thumb tip. Apart from their sensors and actuators, they give a high degree of motion and precision in the hands of robots.

Authors introduced a solution to the problem in [29] of spring damping among rotors and links, which makes state feedback linearization unachievable, is resolved by designing an input-output feedback linearization approach to trajectory tracking control of flexible joint robots (FJRs). The method is validated through simulations and outperforms conventional state feedback linearization techniques. The process breaks through spring damping limitations and can be applied to FJRs, which are widely utilized in industrial, rehabilitative, and aerospace fields.

Including parallel nonlinear and linear controllers, the Data-Driven Inversion-Based Control (IBC) method forms nonlinear control systems with a two-degree-of-freedom structure implemented in [30]. It circumvents the need for comprehensive system knowledge using input/output data and convex optimization. The efficacy is shown using a simulation of a Duffing system in that paper.

A nonlinear output feedback control technique proposed by the authors in [31] that employs state estimation and an algebraic transformation to align with the model’s gain. The minimal-order controller incorporates integrated action and manages output and set points independently. Their efficacy is shown using a simulation of an unstable exothermic Continuous Stirred-Tank Reactor (CSTR).

Integral resonant controllers are implemented in [32] for managing nonlinear vibrations in rotor active magnetic bearing systems. Two controllers mitigate lateral oscillations, with efficacy dependent on control and feedback gains. Stability research and simulations validate that the best design guarantees efficient vibration suppression and linear system performance.

The work in [33] explores set-point control for fully actuated robotic systems using four nonlinear control laws: PD with gravity compensation, PD with desired gravity compensation, computed torque, and augmented PD with gravity compensation. Stability conditions are derived, and simulations with a two-degree-of-freedom manipulator highlight the differences and effectiveness of the controllers.

The three-degrees-of-freedom manipulator uses an in-parallel actuated mechanism proposed by the authors in [34], providing two degrees of rotational flexibility and one degree of translational freedom. The fundamental kinematic equations are formulated, and the influence of physical restrictions on the range of motion is examined. Numerous prospective applications using the in-parallel method are also proposed.

In [35], the authors have implemented the fuzzy immunomodulating PID and FOPID controllers for a three-degree-of-freedom robotic manipulator. The clonal selection technique performs the tuning, and the performance is assessed through mean square error (MSE). The results indicate that the fuzzy immune FOPID controller outperforms others in minimizing tracking errors. The system is run in MATLAB.

The authors in [36] have presented a parallel camera stabilization manipulator with three angular degrees of freedom, regulated by linear actuators to mitigate vehicle disturbances. The system utilizes IMUs for real-time feedback and a Kalman filter for noise attenuation, using inverse kinematics for velocity regulation. Experimental findings demonstrate its efficacy in tracking reference inputs, establishing it as a robust stabilizing solution for terrestrial and airborne vehicles.

The fundamental Artificial Bee Colony (ABC) and the augmented MGABC optimization techniques for optimizing PID controller gains in a 3-DOF manipulator are proposed in [37]. A Lyapunov-based objective function is introduced, demonstrating enhanced efficacy compared to conventional error-based functions in trajectory tracking, disturbance robustness, payload variability, and joint flexibility adaptability. MGABC surpasses ABC in evading local optima, presenting a viable optimization approach for robotic PID controllers.

In [38], the authors have presented the self-tuning fuzzy sliding mode controller (ST-FSMC) for trajectory tracking of a three-degree-of-freedom robotic manipulator. Simulation findings indicate that ST-FSMC reduces the steady-state error to 0.0036 rad, surpassing traditional PID, SMC, and FSMC controllers. The ST-FSMC provides enhanced tracking, resilience, and insensitivity to parameter fluctuations.

A 3D, 4-DOF robotic arm that manipulates items using image processing and inverse kinematics is presented in [39]. A stationary camera identifies the object’s location, and the robot computes the servo arm angles necessary to access the object. Testing revealed errors of 2.58% in object detection, 12.68% in servo motion, and 7.85%, 6.31%, and 12.77% along the x, y, and z axes, respectively. The system exhibits a reliability of 66.66%.

The four-degree-of-freedom (DOF) robotic arm is governed by an Atmega328 microprocessor and Arduino Uno, designed for accuracy in repeated operations. The arm’s performance was assessed for repeatability, precision (180^°^ base, 90^°^ shoulder and elbow, 45^°^ wrist), and payload capacity (0.058 kg). The design is appropriate for educational and small-scale industrial uses [40].

The authors in [41] have proposed sophisticated control techniques for active suspension systems to enhance ride comfort and vehicle handling. Five control approaches are executed, simulated, and evaluated: Proportional-Integral-Derivative (PID) and Linear Quadratic Regulator (LQR). The research assesses the efficacy of each control approach using a comprehensive system model with parameter uncertainty. The results underscore the advantages and drawbacks of the methodologies, indicating that some techniques may not provide stability among all uncertainties.

In [42], the authors introduce a 4-DoF wearable haptic device for the palm to simulate feelings of interaction with inclined surfaces and edges. The apparatus comprises a fixed upper body, a movable end-effector, and articulated arms with four servo motors. The end-effector provides input on pressure, skin stretch, and tangential motion and can fold to replicate various curvatures. The document delineates the device’s design, mobility, statics, kinematics, and a statistically assessed position control mechanism.

Current prosthetic hand controllers face challenges with high degrees of freedom prostheses owing to substantial training data demands and the need for recalibration. A recent 3-DoF controller with ciEMG electrodes and KNN mapping maintains stability without retraining and is implemented in [43] by the authors. Nevertheless, KNN becomes unfeasible with elevated degrees of freedom. A controller that integrates linear interpolation, muscle synergy, and a minimum of two ciEMG channels per degree of freedom is presented for reliable high-degree-of-freedom control.

In [45], GOAL produces comprehensive movements for the torso, hands, and head to facilitate object grabbing. The process requires a 3D object and an initial body position as input, using two networks: GNet for target poses and MNet for motion creation. The technique manages ambulation, cranial alignment, and authentic hand-object interaction. GOAL, trained on the GRAB dataset, surpasses baseline models, demonstrating significant realism in the produced kinematics.

The current robotic grasping and manipulation developments, particularly in the Robotic Grasping and Manipulation Competitions (RGMCs), are implemented in [46]. It offers a summary of historical benchmarks, the design approach for manipulation tasks, and a comprehensive study of the obstacles encountered by competing teams. The paper emphasizes significant challenges and proposes avenues for further research.

After a comprehensive review of the existing literature, the following gaps and challenges have been identified:

- Individuals with physical disabilities often struggle to perform Activities of Daily Living (ADLs) independently and rely on assistive technologies or human support to complete basic tasks.
- Most existing control strategies rely on linear controllers; however, real-world prosthetic systems exhibit significant nonlinearities such as time-varying dynamics and unbalanced loads, which linear methods fail to address adequately.
- There is a noticeable lack of research applying High-Order Fully Actuated Sliding Mode Control (HOFA) to prosthetic systems, particularly in addressing non-linear characteristics such as volatile loads, rapid positional changes, and variable velocities.
- While conventional Sliding Mode Control (SMC) has been explored for prosthetic applications, studies integrating high-order SMC approaches remain scarce, especially regarding handling uncertainties and performance degradation under dynamic conditions.
- The issue of chattering, a well-known limitation of SMC, and the associated stability analysis under varying operating conditions have received insufficient attention in the current body of work.
- There is a lack of comparative evaluations between feedback linearization controllers and high-order sliding mode controllers in unbalanced load conditions—an essential factor in realistic prosthetic hand control scenarios.

This paper proposes the High Order Fully Actuated (HOFA) SMC and FLC controller to compensate for real-world non-linearities or disturbances. A model of a full hand 3DOF thumb and 4DOF index, middle, ring, and pinky finger is examined under different loads and unbalanced conditions. The specific value of forces is applied to each finger depending on the ability of each Robotic finger. Besides the performance of high-order HOFA-SMC with different variations of parameters, we have implemented the Feedback Linearization Controller and compared both controller results with each other and with previous studies. Regarding better performance, HOFA-SMC is superior; it is more robust under uncertainties and achieves fast convergence. By developing a prosthetic model of the entire hand, the study provides a practical answer by highlighting functional aspects such as flexion, extension, and grasping. The traditional SMC has specific challenges, such as chattering issues, which can cause convergence delays and high-frequency oscillations. The controller combines equivalent, sliding, and integral control to achieve high-order tracking. In this paper, after implementing HOFA-SMC, we compared it with FLC, and the results proved the superiority of HOFA-SMC. Moreover, stability analysis is done by implementing the Lyapunov stability theorem. Summarising the author’s contribution:

- The HOFA-SMC is proposed to improve an area of prosthetics in the medical field. This comprehensive idea aims to significantly improve individuals’ overall quality of life by giving them the autonomy to do daily activities and recover essential hand motions.
- Two comparative controllers and three comparative test scenarios have been implemented: the first is flexion, the second is extension, and the third is grasping a ball. All scenarios were done under unbalanced load conditions.
- HOFA-SMC and FLC are specially designed to work under disturbances. This discovery closes a long-standing gap in prosthesis research by using a nonlinear sliding mode control technique developed with the High Order Fully Actuation (HOFA) method and feedback linearization control.
- Stability analysis is evident using the Lyapunov Stability Theorem.
- The proposed controller performs better with more degrees of freedom and covers the paper’s future extension part in [20].
- Moreover, experimental results are compared with literature studies, too. Our hands-on approach, using Python, sets our work apart by increasing accessibility and viability. This method efficiently closes the gap between theoretical concepts and practical implementation, expanding the applicability of our technology.
- Another aspect of this work is the implementation of a feedback linearization controller on a full-hand model. This controller uses feedback from the primary states to achieve the desired stability and offers the advantage of quickly determining the controller gains.
- All parameters used in this study are mentioned in tables S1 Appendix.
- Our hands-on approach, using Python, sets our work apart by increasing accessibility and viability. This method efficiently closes the gap between theoretical concepts and practical implementation, expanding the applicability of our technology.
  Focusing on the technical aspects of methods, such as system actuation, control design, robustness, and performance, allows us to distinguish between core contributions clearly.
  In system actuation, the HOSMC is designed for systems that may not have full actuation, that is, systems with fewer independent control inputs than system states (under-actuated systems) [52]. In contrast, the HOFA-SMC is tailored explicitly for fully actuated systems, where the number of control inputs equals or exceeds the number of system states. This provides complete state-feedback control [53]. In the case of our robotic prosthetic hand with multiple degrees of freedom, such as four degrees of freedom (4-DOF) in each finger and three degrees of freedom (3-DOF) in the thumb, the control inputs (the actuators that apply forces to the joints) correspond directly to the number of joints or degrees of freedom. This ensured that each degree of freedom could be controlled independently, allowing complete feedback control.
  In HOSMC, the controller forces the system trajectory to the sliding surface, and higher-order derivatives of the error are used in the sliding dynamics. It enhances conventional sliding mode control by reducing chattering in systems with higher-order dynamics. In contrast, the HOFA-SMC utilizes a higher-order sliding mode approach combined with a fully actuated control law that ensures independent control of the system states [54]. The sliding mode adapts to the full control inputs, ensuring that the system converges to the desired equilibrium more precisely. In our research, the control input u(t) of the thumb is 7.2, the MCP joint of the thumb, the control input u(t)=6.24, and similarly for other joints, such as the IP joint, the control inputs are calculated based on their respective errors.
  The HOSMC improves disturbance rejection and is robust to system uncertainties and external disturbances using higher-order sliding surfaces. The HOFA-SMC improves upon the HOSMC by explicitly utilizing full actuation to minimize chattering further, enhancing the robustness of the system to both internal uncertainties and external disturbances. In our research, the HOFA-SMC achieves superior disturbance rejection and trajectory tracking, which further minimizes chattering, as seen in the rapid convergence times (under 0.2 s).
  HOSMC can be applied to multi-degree-of-freedom (MDOF) systems; however, its performance may degrade if full actuation is unavailable. In contrast, the HOFA-SMC excels in MDOF systems, where complete state feedback control is required to track and regulate all system states independently. In our study, the movement of each joint was independently regulated to ensure precise and stable tracking of all system states.

The rest of the paper is arranged in the following order: Sect 2 is model design, parameters, and control of HOFA-SMC. Sect 3 covers the system modelling for both controllers, including the controller preliminaries, design, switching actions, state space representation of the proposed model, and the parametric values for both controllers. Sect 4 presents the simulation results for both controllers applied to the full-hand model. Sect 5 compares sliding mode control with feedback linearization control to determine which controller performs better for the full-hand model. This comparison is based on performance results, including the maximum flexion angles of both joints, the time to reach the desired response, the maximum values of the applied gain, and the lambda values for both joints. Additionally, we provide experimental validation, comparing our results with those from the previous study, demonstrating that our solution outperforms the previous one. Along with comparing the two-finger results, we will also compare the outcomes of the two controllers. The key conclusions are included in the latter part, along with ideas on future lines of study to progress this field.

The Index, Middle, Ring, and Little fingers are designed with 4-DOF, while the Thumb has 3-DOF, implemented in the Python environment and evaluated across three test scenarios: flexion, extension, and ball grasping. The findings demonstrate that, between the two controllers, HOFA-SMC proficiently compensates for the hand model across various uncertainties, exhibiting swift trajectory tracking and speedy convergence (sub 0.2 seconds). A comparative examination of both implemented controllers is done and juxtaposed with classic SMCs across several control applications to validate this.

## 2 Model structure and high-order sliding mode control implementation

A Sliding Mode Controller (SMC) is a nonlinear control strategy insensitive to nonlinear systems. It drives the system state onto a specified surface, a sliding surface, and maintains it sliding to achieve the desired dynamics. It uses a discontinuous control law to achieve robustness against uncertainties and disturbances, but the switching can generate high-frequency oscillations, known as chattering. To alleviate these shortcomings, the High-Order Fully Actuated Sliding Mode Controller (HOFA SMC) enhances the conventional SMC by confining the system states and their higher-order derivatives, leading to less chattering and smoother control. HOFA SMC, which is developed for fully actuated systems with an independent control input for every degree of freedom, provides enhanced accuracy and robustness, as is needed for high-order systems. These sophisticated systems need resilience along with perfect control performance.

The advantages are particularly pronounced in completely regulated systems with an SMC of high-order sliding mode:

**Robustness to Uncertainties:** After entering the sliding phase, the sliding mode guarantees that external disturbances or uncertainties do not influence its performance.

**Higher-Order Harmonics Elimination:** The controller reduces the order of differential equations describing the system and eliminates the effect of higher frequency signals, thus simplifying the system’s dynamics.

This method guarantees accurate control and stability even with large nonlinearities and uncertainties.

### 2.1 Preliminaries of HOFA-SMC

**Fig 1:**
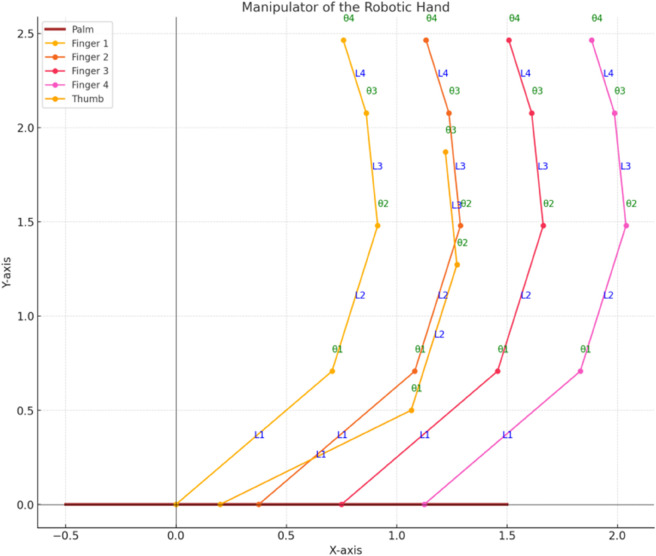
Manipulator of the robotic hand. Manipulator is showing the configuration of the joints and the corresponding generalized coordinates for the 4-DOF system.

Lagrangian Formulation of Dynamics for a 4-DOF Robotic Hand Defining a set of generalised coordinates $p\in {\mathbb{R}}^{n}$ that characterises the system’s configuration comes first in formulating Lagrangian dynamics. Fig 1 shows the manipulator of the robotic hand. For a 4-DOF planar robotic hand, the generalized coordinates are the joint angles $\alpha =[\alpha_{1},\alpha_{2},\alpha_{3},\alpha_{4}{]}^{T}$, where each $\alpha_{i}$ represents the rotational position of a joint in the hand.

Once the generalized coordinates are chosen, we define the generalized forces $\tau \in {\mathbb{R}}^{n}$, representing the torques applied at each joint. The Lagrangian function $𝒦(p,\dot{p})$ is defined as the difference between the system’s total kinetic energy $E(p,\dot{p})$ and the potential energy *V*(*p*) shown in 1:

$$𝒦(p,\dot{p})=E(p,\dot{p})-V(p)$$ (1)

The equations of motion are derived using the Euler-Lagrange model given by 2:

$$\frac{d}{dt}\left(\frac{\partial 𝒦}{\partial {\dot{p}}_{i}}\right)-\frac{\partial 𝒦}{\partial p_{i}}=\tau_{i}-\frac{\partial ℛ}{\partial {\dot{p}}_{i}}$$ (2)

Where ℛ represents the Rayleigh dissipation function, capturing energy dissipation (e.g., damping effects), defined as 3:

$$ℛ=\sum_{i=1}^{n}\frac{1}{2}\zeta_{i}{\dot{\alpha }}_{i}^{2}$$ (3)

Here, $\zeta_{i}$ represents the damping coefficient for each joint, which accounts for natural resistance to movement and helps to reduce oscillations.

For a 4-DOF planar robotic hand, the center of mass of each finger segment can be described in terms of its position coordinates $\left(u_{i},v_{i}\right)$, derived from the joint angles. The position of the center of mass of each segment is given by 4:

$$u_{i}=d_{i}\cos\left(\sum_{k=1}^{i}\alpha_{k}\right),\;v_{i}=d_{i}\sin\left(\sum_{k=1}^{i}\alpha_{k}\right)$$ (4)

Where *d*_*i*_ is the distance from the joint to the center of mass of segment *i*.

By differentiating these position expressions concerning time, the linear velocities ${\dot{x}}_{i}$ and ${\dot{y}}_{i}$ are derived. This results in the derivation of kinetic energy, which includes both translational and rotational components. The height of each segment’s center of mass about a fixed reference frame—which is affected by gravity—determines the potential energy *U*.

Finally, the system dynamics are expressed in matrix form as written in eq. 5:

$$\tau =M(\alpha )\ddot{\alpha }+C(\alpha ,\dot{\alpha })\dot{\alpha }+G(\alpha )$$ (5)

Here, the inertia matrix captures the mass distribution and segment lengths, represented by M(α). The Coriolis and the centripetal matrix is represented by $C(\alpha ,\dot{\alpha })$, which adds hypothetical forces and contains terms reliant on joint velocities. Gravitational influences on each joint are considered by the gravitational force vector, G(α).

By means of controlled and modelled behaviour of every joint under applied torques, this method allows exact simulation of the dynamics of the 4-DOF robotic hand. The developed equations provide a strong basis for trajectory planning and force control in robotic hand applications.

The sizes and limitations of the application dictate the lengths of the links of a robotic hand manipulator, which is composed of four degrees of freedom in the fingers and three in the thumb; these are typically based on human proportions. The distal phalanx (tip segment) is 15 to 30 mm, the middle phalanx (middle knuckle to distal knuckle) is 20 to 40 mm, and the proximal phalanx (base to middle knuckle) is 30 to 50 mm. For the thumb, the proximal phalanx is 20–30 mm, the distal phalanx is 15–25 mm, and the metacarpal segment (base to knuckle) is 20–40 mm. The radius of the palm that supports the thumb and fingers is normally between 60 and 100 mm.

The lengths may be augmented for heavy-duty applications or reduced for precision tasks. Design considerations must ensure the thumb has enough workspace, dexterity, and opposability to integrate material constraints, actuation, and control accuracy. In precision applications, shorter links are favoured as they enhance control and necessitate reduced torque. Prototyping and simulations must be used to refine these first characteristics for the specific use case.

### 2.2 High-order sliding mode control for force control in fully actuated systems

In this paper, we present a High-Order Sliding Mode (HOSM) Controller for regulating the force applied by robotic fingers. The controller combines equivalent control, sliding mode control, and integral control to achieve desired force tracking while minimising chattering. We model the system as fully actuated, where the control input directly impacts the force dynamics.

### 2.3 System dynamics and control law

Let the following state variables govern the system: $\vec{x}(t)$ is the Measured force (position variable) of the robotic finger. $\dot{\vec{x}}(t)$ is the measured force derivative (velocity). $\vec{e}(t)={\vec{x}}_{\text{d}}\;-\;\vec{x}(t)$ is the error in force . $\dot{\vec{e}}(t)={\dot{\vec{x}}}_{\text{d}}-\dot{\vec{x}}(t)$: is the error in velocity.

The objective of the controller is to reduce the error $\vec{e}(t)$ to zero over time, ensuring that the force $\vec{x}(t)$ tracks the desired force ${\vec{x}}_{\text{d}}$.

The sliding surface $\vec{s}(t)$ is defined in 6:

$$\vec{s}(t)=\dot{\vec{x}}(t)+\lambda \vec{e}(t)$$ (6)

Here, λ is a sliding mode parameter.

The equivalent control law ${\vec{u}}_{\text{eq}}(t)$ is the ideal control input that eliminates the sliding mode dynamics as shown in 7:

$${\vec{u}}_{\text{eq}}(t)=K_{p}\vec{e}(t)+K_{d}\dot{\vec{e}}(t)+\lambda \vec{e}(t)$$ (7)

Here *K*_*p*_ is the proportional gain, *K*_*d*_ is the derivative gain, and λ is the sliding mode parameter.

To drive the system towards the sliding surface, a saturated switching control ${\vec{u}}_{\text{sw}}(t)$ is introduced in 8:

$${\vec{u}}_{\text{sw}}(t)=-K\;\tanh\left(\frac{\vec{s}(t)}{\delta }\right)$$ (8)

Here, *K* is the sliding mode gain. δ is the boundary layer parameter.

An integral control component *u*_*I*_(*t*) is added to account for steady-state errors as shown in 9:

$$u_{I}(t)=K_{i}\int e(t)\;dt$$ (9)

Here, *K*_*i*_ is the integral gain.

The total control input $\vec{u}(t)$ is a combination of the equivalent control, switching control, and integral control components as shown in 10:

$$\vec{u}(t)={\vec{u}}_{\text{eq}}(t)+{\vec{u}}_{\text{sw}}(t)+{\vec{u}}_{I}(t)$$ (10)

Substituting the expressions for each component to get 11:

$$\vec{u}(t)=\left(K_{p}\vec{e}(t)+K_{d}\dot{\vec{e}}(t)+\lambda \vec{e}(t)\right)+\left(-K\;\tanh\left(\frac{\vec{s}(t)}{\delta }\right)\right)+\left(K_{i}\int \vec{e}(t)\;dt\right)$$ (11)

In a fully actuated system, the control input $\vec{u}(t)$ directly influences the force. The system dynamics can be described as 12:

$$\dot{\vec{x}}(t)=f(\vec{x}(t),\vec{u}(t))$$ (12)

In the simplified model, we approximate the force change as proportional to the control input, we get 13:

$$\dot{\vec{x}}(t)=\alpha \;\vec{u}(t)$$ (13)

Where α is a constant related to the system’s dynamics (e.g., mass, damping, or stiffness).

The High-order (SMC) method applied to each joint of the thumb (3 DOF) and index finger (4 DOF) allows for flexion and extension. The control input for each Middle, Ring, Index, and Pinky joint is calculated by adjusting the joint angles according to the SMC law, ensuring the hand moves to the desired position during flexion (collapsing) and extension (releasing).

### 2.4 Flexion and extension using high-order sliding mode control

The thumb (3-DOF) and index finger (4-DOF) joints are subjected to the High-Order Fully Actuated Sliding Mode Control HOFA-SMC method to experience flexion and extension. The control inputs of the other fingers—the middle, ring, index, and pinky—are calculated through the adjustment of joint angles by the HOFA-SMC law to guarantee accurate motion during extension (releasing) and flexion (grasping). This section provides the control law of such movements, the optimal angles under flexion, and the initial joint angles.

### 2.5 Control parameters

The following parameters are used for HOFA-SMC:


$$K_{p}=10,\;K_{d}=7,\;K_{i}=5,\;\lambda =6,\;K=10,\;\delta =0.05$$

Where: $K_{p},K_{d},K_{i}$: Proportional, derivative, and integral gains. λ: Sliding mode parameter. *K*: Sliding mode gain. δ: Boundary layer thickness for chattering reduction.

### 2.6 HOFA-SMC control law

The control input for each joint is determined using the HOFA-SMC law defined as 14:

$$\vec{u}(t)=K_{p}\vec{e}(t)+K_{d}\dot{\vec{e}}(t)+\lambda \vec{e}(t)-K\;\tanh\left(\frac{\vec{s}(t)}{\delta }\right)+K_{i}\int \vec{e}(t)\;dt$$ (14)

Where: $\vec{e}(t)=\theta_{\text{desired}}\;-\;\theta (t)$: Error between desired and current angles. $\dot{\vec{e}}(t)$: Derivative of error. $\vec{s}(t)=\dot{\vec{e}}(t)+\lambda \vec{e}(t)$: Sliding surface.

### 2.7 Thumb control input: CMC joint dynamics

For the CMC joint of the thumb:


$$\theta_{\text{initial}}=0.5,\;\theta_{\text{desired}}=3.0$$

$$\vec{e}(t)=\theta_{\text{desired}}-\theta_{\text{initial}}=3.0-0.5=2.5$$

$$\dot{\vec{e}}(t)\approx \vec{e}(t)=2.5,\;\vec{s}(t)=\dot{\vec{e}}(t)+\lambda \vec{e}(t)=2.5+1.0(2.5)=5.0$$

Substituting into the control law:


$$\vec{u}(t)=K_{p}\vec{e}(t)+K_{d}\dot{\vec{e}}(t)+\lambda \vec{e}(t)-K\;\tanh\left(\frac{\vec{s}(t)}{\delta }\right)$$

Substitute values:


$$\vec{u}(t)=1.5(2.5)+0.5(2.5)+1.0(2.5)-0.3\;\tanh\left(\frac{5.0}{0.05}\right)$$

$$\vec{u}(t)=3.75+1.25+2.5-0.3(1)$$

$$\vec{u}(t)=7.5-0.3=7.2$$

$$\vec{u}(t)=7.2$$ (15)

The CMC joint is controlled with $\vec{u}(t)=7.2$ as an input from 15. The same is calculated for thumb MCP and IP joints and both joints of the index finger as well. The HOFA-SMC technique ensures bumpless and accurate joint motion during extensions and flexions with less chattering and keeps it stable. The technique is illustrated with numerical computation for major joints to establish its ability to reach target points with accuracy.

Higher-order Fully Actuated Sliding Mode Control (HOFA SMC) follows system dynamics according to the higher-order sliding surfaces to achieve greater robustness and chattering reduction. The family $\sigma_{i}(t)$, with order *i*, is the general form of an *n*-dimensional state-variable system controller. Higher-order sliding mode rules ensure system convergence on a sliding surface because it specifies the control input $\vec{u}(t)$. Sliding surfaces are given as 16,17, and 18:

$$\sigma_{1}=\dot{\vec{e}}+\mu_{1}\cdot \vec{e}$$ (16)

$$\sigma_{2}=\ddot{\vec{e}}+\mu_{2}\cdot \dot{\vec{e}}+\mu_{3}\cdot \vec{e}$$ (17)

$$\sigma_{3}=\dddot{\vec{e}}+\mu_{4}\cdot \ddot{\vec{e}}+\mu_{5}\cdot \dot{\vec{e}}+\mu_{6}\cdot \vec{e}$$ (18)

Where $\vec{e}$ is the tracking error, and $\mu_{i}$ are design parameters for each order of the sliding surface. The control input $\vec{u}(t)$ is then computed by 19:

$$\vec{u}(t)=-K_{1}\cdot \text{sign}(\sigma_{1})-K_{2}\cdot \text{sign}(\sigma_{2})-K_{3}\cdot \text{sign}(\sigma_{3})+\text{adaptive gains}$$ (19)

**Fig 2:**
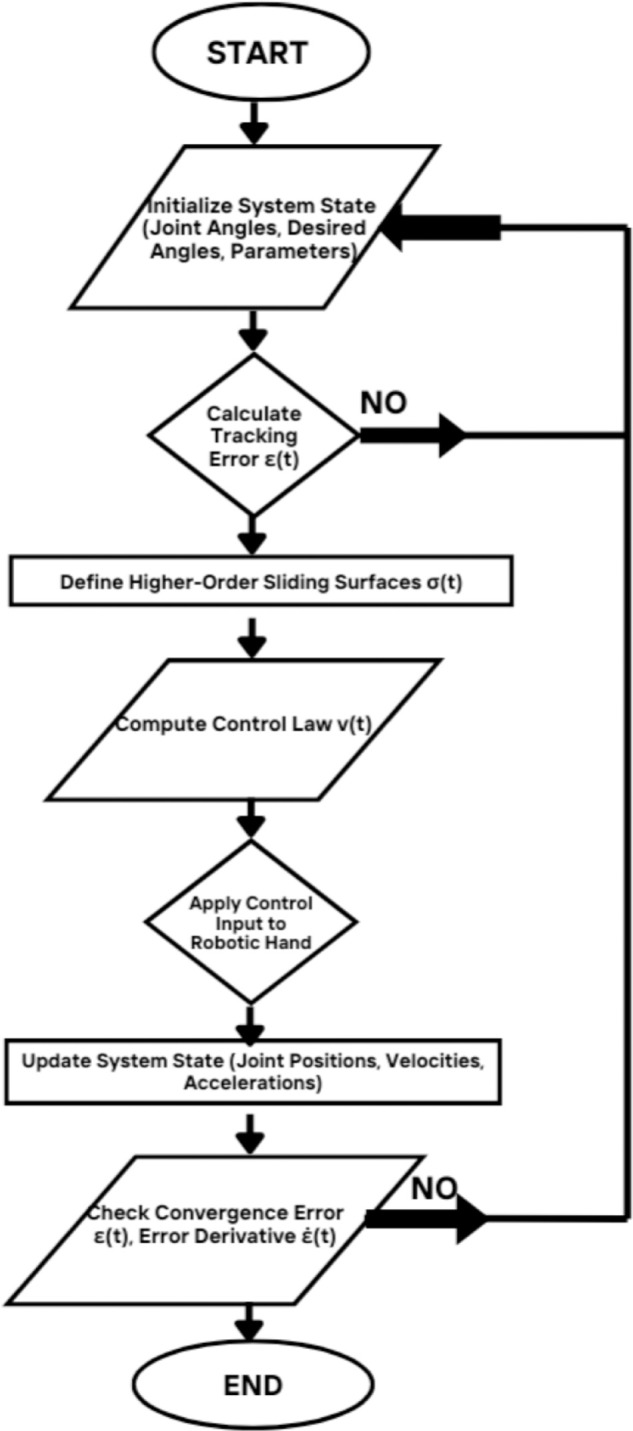
Flowchart of HOFA-SMC strategy. The flowchart illustrates the process of the (HOFA-SMC) strategy for robotic hand control, detailing steps such as system initialization, error calculation, control law computation, and convergence error checking.

The adaptive gain terms $\gamma_{1},\gamma_{2},\ldots$ are introduced to adjust the controller’s real-time performance based on system dynamics. The goal is to provide smooth convergence to the sliding surface while minimizing the chattering effect. The adaptive gains enable real-time changes to efficiently handle uncertainties and disturbances, while the sliding surfaces $\sigma_{1},\sigma_{2},\sigma_{3}$ provide robust tracking of the system’s state variables. The controller ensures that the system converges to the intended trajectory with the least amount of chatter by adjusting these parameters. Fig 2 shows the Flowchart of HOFA-SMC strategy.

### 2.8 Dynamics of extension

The required joint angles are raised to simulate the fingers opening or straightening to accomplish extension. The full extension (π radians, 180^°^) is achieved by calculating the control inputs for the thumb (3 DOF) and index finger (4 DOF) joints using High-Order Fully Actuated Sliding Mode Control (HOFA-SMC).

### 2.9 Control inputs for thumb joint movements

**CMC Joint:** *Initial:* θ=0.7854, *Desired:* $\theta_{\text{desired}}=0.8029$


$$\vec{e}(t)=0.0175,\;\vec{s}(t)=0.0350$$

$$\vec{u}(t)=1.5(0.0175)+0.5(0.0175)+1.0(0.0175)-0.3\;\tanh\left(\frac{0.0350}{0.05}\right)$$

$$\vec{u}(t)=0.1287$$ (20)

**MCP Joint:** *Initial:* θ=0.5236, *Desired:* $\theta_{\text{desired}}=\pi$


$$\vec{e}(t)=2.61799,\;\vec{s}(t)=2.61799$$

$$\vec{u}(t)=1.5(2.61799)+1.0(2.61799)-0.3\;\tanh\left(\frac{2.61799}{0.05}\right)$$

$$\vec{u}(t)=6.24497$$ (21)

**IP Joint:** *Initial:* θ=0.3927, *Desired:* $\theta_{\text{desired}}=\pi$


$$\vec{e}(t)=2.7489,\;\vec{s}(t)=2.7489$$

$$\vec{u}(t)=1.5(2.7489)+1.0(2.7489)-0.3\;\tanh\left(\frac{2.7489}{0.05}\right)$$

$$\vec{u}(t)=6.57225$$ (22)

Here, 20,21,22 are calculated control inputs for thumb 3DOF, which contains three joints. Control inputs are derived to achieve precise motion control for each joint. Ensuring the thumb’s path corresponds with the intended grasping or manipulation.

### 2.10 Control inputs for index finger joints

**MCP Joint:** *Initial:* θ=0.5236, *Desired:* $\theta_{\text{desired}}=\pi$


$$\vec{e}(t)=2.61799,\;\vec{s}(t)=2.61799$$

$$\vec{u}(t)=6.24497$$ (23)

**PIP Joint:** *Initial:* θ=0.3927, *Desired:* $\theta_{\text{desired}}=\pi$


$$\vec{e}(t)=2.7489,\;\vec{s}(t)=2.7489$$

$$\vec{u}(t)=6.57225$$ (24)

**DIP Joint:** *Initial:* θ=0.3142, *Desired:* $\theta_{\text{desired}}=\pi$


$$\vec{e}(t)=2.8274,\;\vec{s}(t)=2.8274$$

$$\vec{u}(t)=6.7685$$ (25)

**IP Joint:** *Initial:* θ=0.2618, *Desired:* $\theta_{\text{desired}}=\pi$


$$\vec{e}(t)=2.8796,\;\vec{s}(t)=2.8796$$

$$\vec{u}(t)=6.9194$$ (26)

The HOFA-SMC method provides accurate control inputs 23,24,25, and 26 for 4-DOF which contains four joints. Ensuring smooth and stable operation of each joint. This approach minimizes chattering and maintains precise trajectory tracking.

We explored several systematic approaches for the selection of *λ*; one such method involved using sensitivity analysis to observe the relationship between *λ* and system performance. By varying *λ* within a predefined range selected from the paper [20,44] and evaluating the resulting performance metrics, we determined an optimal value of *λ* that minimized the tracking error while ensuring stability across all test scenarios 27,28.

It began by introducing the sliding surface, a crucial component of our control strategy. The sliding surface is given by 27 from [20]:

$$p=\left[\tilde{\dot{x}}+\lambda \right]\tilde{x}\;$$ (27)

This sliding surface governs the system’s position and velocity control. It establishes a relationship between the tracking error and the sliding mode that we intend to achieve. Then, a relationship was derived between the derivative of the sliding surface, the system dynamics, and the control input is given by 28 from [20]:

$$\hat{i}=-f+\ddot{x_{d}}-\lambda \tilde{\dot{x}}\;$$ (28)

In this equation, *λ* is a key parameter in the control law, affecting the system’s stability and tracking accuracy by influencing the derivative of the tracking error.

### 2.11 System setup and force requirements

In the simulated hand model, forces are distributed across the thumb, index, middle, ring, and little fingers to emulate a stable grasp. The force requirements and their distribution are derived from the object’s geometry (a sphere with a radius of 10 cm) and weight. The applied forces are as follows: Thumb: 1.53N , Index: 1.53N , Middle: 1.23N, Ring: 0.92N and Little: 0.92N. The forces are applied tangentially to the sphere’s surface, ensuring a stable grasp. 29 used to calculate the gravitational force.

### 2.12 For grasping

- **Sphere Radius (*R*)**: 12cm
- **Object Weight (*W*)**: 2kg
- **Gravitation Force (F**_***g***_)
  $${\vec{F}}_{g}=W\cdot g$$ (29)
=2·9.81=19.62N
- **Coefficient of Friction (μ)**: 0.6
- **Forces Applied**:
  Thumb: 1.53N, Index: 1.53N, Middle: 1.23N, Ring: 0.92N and Little: 0.92N

### Contact geometry

The coordinates of each finger’s contact point are calculated using 30:

$$x_{f}=R\cos(\theta_{f}),\;y_{f}=R\sin(\theta_{f})$$ (30)

Angular positions ($\theta_{f}$) for the fingers:

Thumb: $45^{\circ }$, Index: $90^{\circ }$, Middle $135^{\circ }$, Ring: $180^{\circ }$ and Little: $225^{\circ }$

**Contact Points**


$$x_{\text{thumb}}=R\cos(45^{\circ })=12\cdot 0.707=8.49\;\text{cm}$$

$$y_{\text{thumb}}=R\sin(45^{\circ })=12\cdot 0.707=8.49\;\text{cm}$$

$$x_{\text{index}}=R\cos(90^{\circ })=12\cdot 0=0\;\text{cm}$$

$$y_{\text{index}}=R\sin(90^{\circ })=12\cdot 1=12\;\text{cm}$$

$$x_{\text{middle}}=R\cos(135^{\circ })=12\cdot (-0.707)=-8.49\;\text{cm}$$

$$y_{\text{middle}}=R\sin(135^{\circ })=12\cdot 0.707=8.49\;\text{cm}$$

$$x_{\text{ring}}=R\cos(180^{\circ })=12\cdot (-1)=-12\;\text{cm}$$

$$y_{\text{ring}}=R\sin(180^{\circ })=12\cdot 0=0\;\text{cm}$$

$$x_{\text{little}}=R\cos(225^{\circ })=12\cdot (-0.707)=-8.49\;\text{cm}$$

$$y_{\text{little}}=R\sin(225^{\circ })=12\cdot (-0.707)=-8.49\;\text{cm}$$

### Force calculations

The tangential forces (*F*_*t*_) are calculated using 31:

$${\vec{F}}_{t}=\mu \cdot {\vec{F}}_{n}$$ (31)

For each finger:


$${\vec{F}}_{t}^{\text{thumb}}=0.6\cdot 1.53=0.918\;\text{N}$$

$${\vec{F}}_{t}^{\text{index}}=0.6\cdot 1.53=0.918\;\text{N}$$

$${\vec{F}}_{t}^{\text{middle}}=0.6\cdot 1.23=0.738\;\text{N}$$

$${\vec{F}}_{t}^{\text{ring}}=0.6\cdot 0.92=0.552\;\text{N}$$

$${\vec{F}}_{t}^{\text{little}}=0.6\cdot 0.92=0.552\;\text{N}$$

The total tangential forces must counteract the object’s weight 32:

$$\sum {\vec{F}}_{t}={\vec{F}}_{t}^{\text{thumb}}+{\vec{F}}_{t}^{\text{index}}+{\vec{F}}_{t}^{\text{middle}}+{\vec{F}}_{t}^{\text{ring}}+{\vec{F}}_{t}^{\text{little}}$$ (32)

$$\sum {\vec{F}}_{t}=0.918+0.918+0.738+0.552+0.552=3.678\;\text{N}$$

The total applied force which is (3.678N) is calculated from 32.

## 3 Model structure, design, and control of feedback linearization control

A popular method for controlling nonlinear systems is feedback linearization, which provides a way to construct controllers without depending on traditional linearization around specific operating points [47]. This technology converts a nonlinear system into an equivalent linear representation using a change of variables. Creating a control input that creates a linear input-output mapping for the final linearized system is the main goal [48].

This method allows for the regulation of inherently nonlinear processes by transforming nonlinear dynamics into a linear structure within a specific operating range. The majority of real-world systems are nonlinear. Hence, feedback linearization offers substantial benefits regarding process regulation and robustness. It improves the overall control strategy by reducing the complexity of determining controller gains and achieving stability through feedback from the system’s primary states.

This section presents a Feedback Linearization Controller (FLC) designed to regulate the force applied by robotic fingers. The system is modeled as fully actuated, where the control input directly influences the force dynamics.

$$\vec{\tau }=M(\alpha )\ddot{\alpha }+C(\alpha ,\dot{\alpha })\dot{\alpha }+G(\alpha )$$ (33)

Here in 33 M(α) is the inertia matrix, capturing the mass distribution and segment lengths. $C(\alpha ,\dot{\alpha })$ is the Coriolis and centripetal matrix, which includes terms dependent on joint velocities and introduces fictitious forces. G(α) is the gravitational force vector, accounting for gravitational effects on each joint.

### 3.1 4-DOF finger dynamics

**Inertia matrix**

The elements of the inertia matrix M(α) are computed for the 4-DOF finger as 34:


$$M_{ij}=\text{Entries depend on joint pairs }(\alpha_{i},\alpha_{j}),\;i,j\in \{1,2,3,4\}.$$

For Fingers:

$$M_{11}=\frac{m_{1}l_{1}^{2}}{3}+m_{2}l_{1}^{2}+m_{3}(l_{1}^{2}+\frac{l_{2}^{2}}{4})+m_{4}(l_{1}^{2}+l_{2}^{2}+l_{3}^{2})+m_{t}(l_{1}^{2}+l_{2}^{2}+l_{3}^{2}+l_{4}^{2})+\sum_{k=2}^{4}m_{k}l_{1}l_{k}\cos(\alpha_{k})$$ (34)

### Coriolis and centripetal forces 35

$$C_{i}=\sum_{j=1}^{4}\sum_{k=1}^{4}C_{ijk}(\alpha ,\dot{\alpha }){\dot{\alpha }}_{j}{\dot{\alpha }}_{k}$$ (35)

For Fingers:


$$C_{1}=-\sum_{k=2}^{4}(m_{k}l_{1}l_{k}{\dot{\alpha }}_{1}{\dot{\alpha }}_{k}\sin\alpha_{k})$$

### Gravitational forces 36

$$G_{i}=\sum_{j=1}^{4}m_{j}l_{j}g\sin(\alpha_{1}+\alpha_{2}+\alpha_{3}+\alpha_{4})$$ (36)

### 3.2 3-DOF thumb dynamics


$$M_{11}=\frac{m_{1}l_{1}^{2}}{3}+m_{2}l_{1}^{2}+m_{3}(l_{1}^{2}+\frac{l_{2}^{2}}{4})+m_{t}(l_{1}^{2}+l_{2}^{2}+l_{3}^{2})+\sum_{k=2}^{3}m_{k}l_{1}l_{k}\cos(\alpha_{k})$$

### Coriolis and centripetal forces


$$C_{1}=-\sum_{k=2}^{3}(m_{k}l_{1}l_{k}{\dot{\alpha }}_{1}{\dot{\alpha }}_{k}\sin\alpha_{k})$$

### Gravitational forces


$$G_{i}=\sum_{j=1}^{3}m_{j}l_{j}g\sin(\alpha_{1}+\alpha_{2}+\alpha_{3})$$

### 3.3 Preliminaries of feedback linearization

The dynamics of a 4-DOF finger and a 3-DOF thumb can be expressed using the Lagrange-Euler dynamic model 37:

$$M(\alpha )\ddot{\alpha }+F(\alpha ,\dot{\alpha })\dot{\alpha }+G(\alpha )=\tau$$ (37)

Where:

- $\vec{\alpha }=[\alpha_{1},\alpha_{2},\ldots ,\alpha_{n}{]}^{T}$ is the joint angle vector for the 4-DOF fingers, and $\vec{\alpha }=[\alpha_{1},\alpha_{2},\alpha_{3}{]}^{T}$ for the 3-DOF thumb.
- $\vec{\tau }=[\tau_{1},\tau_{2},\ldots ,\tau_{n}{]}^{T}$ represents the torques for the finger, and $\vec{\tau }=[\tau_{1},\tau_{2},\tau_{3}{]}^{T}$ for the thumb.
- M(α) is the symmetric inertia matrix ($M_{ij}=M_{ji}$).
- $F(\alpha ,\dot{\alpha })\dot{\alpha }$ accounts for Coriolis and centrifugal forces.
- G(α) represents gravitational forces.

### 3.4 Finger (4-DOF) dynamics

#### Inertia Matrix 38:

$$M(\alpha )=[\begin{array}{cccc} M_{11} & M_{12} & M_{13} & M_{14}\\ M_{21} & M_{22} & M_{23} & M_{24}\\ M_{31} & M_{32} & M_{33} & M_{34}\\ M_{41} & M_{42} & M_{43} & M_{44} \end{array}]$$ (38)

Each element *M*_*ij*_ depends on the link masses, lengths, and coupling terms.

#### Coriolis and Centripetal Forces 39:

$$\vec{F}(\alpha ,\dot{\alpha })\dot{\alpha }=[\begin{array}{c} F_{1}(\alpha ,\dot{\alpha })\\ F_{2}(\alpha ,\dot{\alpha })\\ F_{3}(\alpha ,\dot{\alpha })\\ F_{4}(\alpha ,\dot{\alpha }) \end{array}]$$ (39)

#### Gravitational Forces 40:

$$\vec{G}(\alpha )=[\begin{array}{c} G_{1}(\alpha )\\ G_{2}(\alpha )\\ G_{3}(\alpha )\\ G_{4}(\alpha ) \end{array}]$$ (40)

### 3.5 State-space representation

The state-space representation for both systems is the following 41:

$$[\begin{array}{c} {\dot{X}}_{1}\\ {\dot{X}}_{2} \end{array}]=[\begin{array}{c} X_{2}\\ P \end{array}]+[\begin{array}{c} O_{n\times n}\\ Q \end{array}]\vec{\tau }$$ (41)

Where:

- *n* = 4 for the finger, *n* = 3 for the thumb.
- $\vec{P}=-M^{-1}(\alpha )\left[\vec{F}(\alpha ,\dot{\alpha })\dot{\alpha }+\vec{G}(\alpha )\right]$.
- $\vec{Q}=M^{-1}(\alpha )$.
- ${\vec{X}}_{1}=[\alpha_{1},\alpha_{2},\ldots ,\alpha_{n}{]}^{T},\;{\vec{X}}_{2}=[{\dot{\alpha }}_{1},{\dot{\alpha }}_{2},\ldots ,{\dot{\alpha }}_{n}{]}^{T}$.

### 3.6 Feedback linearization control law

We define the following 42 to linearize the system.

$$\vec{P}+\vec{Q}\vec{\tau }=v$$ (42)

This implies 43:

$$\vec{\tau }=-{\vec{Q}}^{-1}\vec{P}+{\vec{Q}}^{-1}v$$ (43)

### 3.7 Desired torque and stability

Using the desired torque ${\vec{\tau }}_{d}$ and control vector $v_{d}$ 44:

$${\vec{\tau }}_{d}={\vec{Q}}^{-1}(-\vec{P}+v_{d})$$ (44)

Substituting ${\vec{\tau }}_{d}$ we get 45:

$$\ddot{Y}=v=\vec{P}+\vec{Q}({\vec{Q}}^{-1}(-\vec{P}+v_{d}))$$ (45)

### 3.8 Error dynamics

To account for modeling errors ε:


$$M(\alpha )\ddot{\alpha }+\vec{F}(\alpha ,\dot{\alpha })\dot{\alpha }+\vec{G}(\alpha )+\varepsilon =\vec{\tau }$$

With feedback linearization we get 46:

$$\ddot{\alpha }=v+M^{-1}(\alpha )\varepsilon$$ (46)

### 3.9 Linearized system

Feedback linearization transforms linear dynamics into 47:

$$\ddot{\alpha }=v+M^{-1}(\alpha )\varepsilon$$ (47)

**Fig 3:**
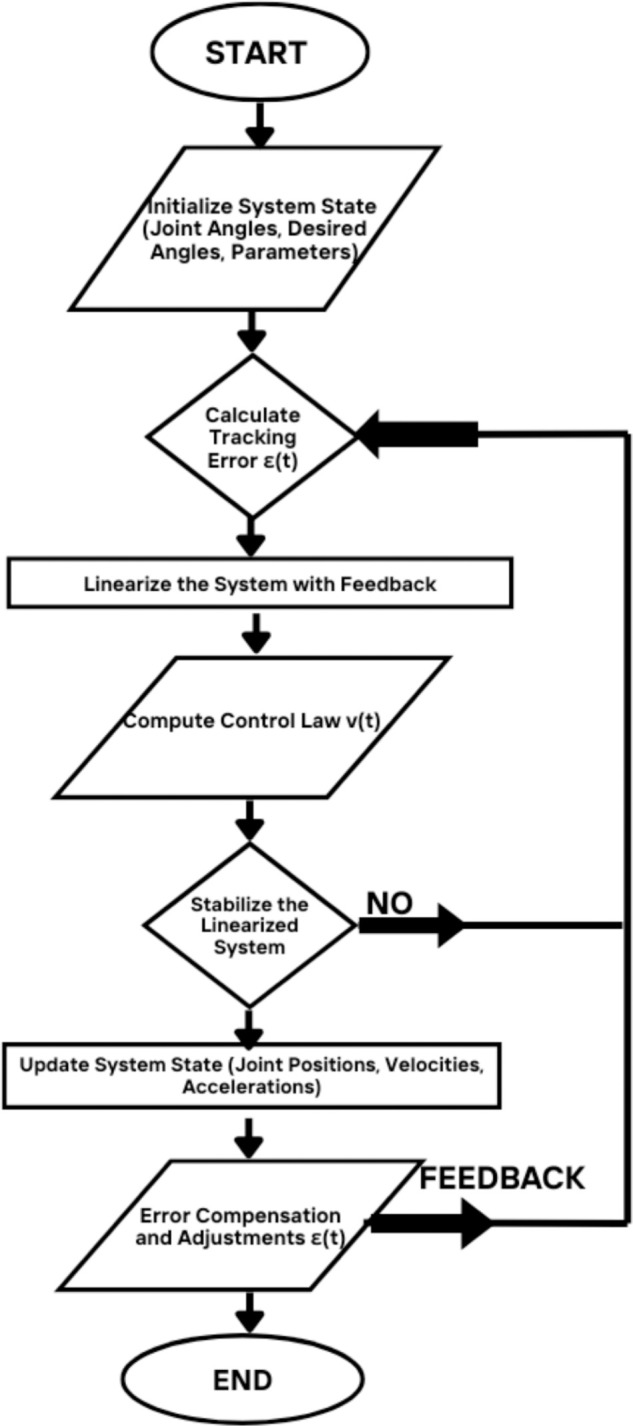
Flowchart of FLC strategy. The flowchart illustrates the step-by-step process for implementing a (FLC) strategy, including system initialization, error calculation, feedback linearization, control law computation, and system stabilization.

Where *v* is the feedback linearization control input, ensuring robust and stable system performance. All parametric values are in S1 Appendix. Fig 3 shows the Flowchart of FLC strategy.

### 3.10 MAE calculations for proposed controller

- Number of data points: *n* = 4
- Reference values (${\vec{y}}_{i}$) and Predicted values (${\hat{\vec{y}}}_{i}$) for each joint:
  – **Joint *q***_**1**_: ${\vec{y}}_{i}=[2.0,2.2,2.4,2.6]$, ${\hat{\vec{y}}}_{i}=[2.01,2.19,2.39,2.59]$
  – **Joint *q***_**2**_: ${\vec{y}}_{i}=[1.0,1.2,1.4,1.6]$, ${\hat{\vec{y}}}_{i}=[1.01,1.19,1.38,1.59]$
  – **Joint *q***_**3**_: ${\vec{y}}_{i}=[0.5,0.7,0.9,1.1]$, ${\hat{\vec{y}}}_{i}=[0.51,0.69,0.88,1.09]$

**Calculations**

#### Joint *q*_1_:


$$MAE_{q_{1}}=\frac{1}{n}\sum_{i=1}^{n}|{\vec{y}}_{i}-{\hat{\vec{y}}}_{i}|$$

$$=\frac{1}{4}\left(|2.0-2.01|+|2.2-2.19|+|2.4-2.39|+|2.6-2.59|\right)$$

$$=\frac{1}{4}\left(0.01+0.01+0.01+0.01\right)=\frac{0.04}{4}=0.01$$

#### Joint *q*_2_:


$$MAE_{q_{2}}=\frac{1}{n}\sum_{i=1}^{n}|{\vec{y}}_{i}-{\hat{\vec{y}}}_{i}|$$

$$=\frac{1}{4}\left(|1.0-1.01|+|1.2-1.19|+|1.4-1.38|+|1.6-1.59|\right)$$

$$=\frac{1}{4}\left(0.01+0.01+0.02+0.01\right)=\frac{0.05}{4}=0.0125$$

#### Joint *q*_3_:


$$MAE_{q_{3}}=\frac{1}{n}\sum_{i=1}^{n}|{\vec{y}}_{i}-{\hat{\vec{y}}}_{i}|$$

$$=\frac{1}{4}\left(|0.5-0.51|+|0.7-0.69|+|0.9-0.88|+|1.1-1.09|\right)$$

$$=\frac{1}{4}\left(0.01+0.01+0.02+0.01\right)=\frac{0.05}{4}=0.0125$$

### Proposed values for MAE

- Joint *q*_1_: $MAE_{q_{1}}=0.01\;\text{rad}$
- Joint *q*_2_: $MAE_{q_{2}}=0.0125\;\text{rad}$
- Joint *q*_3_: $MAE_{q_{3}}=0.0125\;\text{rad}$

### 3.11 IAE calculations for proposed controller

Formula for IAE The Integral Absolute Error (IAE) is calculated as:


$$IAE=\int_{0}^{T}|e(t)|dt$$

For discrete data:


IAE≈Δt∑|e(t)|

Where:

- |e(t)|: Absolute error at each time step.
- Δt: Time interval between samples.
- T=n·Δt: Total observation time.

**Calculations for Each Joint**

#### Joint *q*_1_.

Given:

- Absolute error values: |e(t)|=[0.02,0.03,0.01,0.04]
- Time interval (Δt): 0.1s
- Number of points: *n* = 4

The IAE formula becomes:


$$IAE_{q_{1}}\approx \Delta t\sum |e(t)|$$

$$IAE_{q_{1}}=0.1\cdot (0.02+0.03+0.01+0.04)$$

$$IAE_{q_{1}}=0.1\cdot 0.10=0.01\;\text{rad}$$

#### Joint *q*_2_.

Given:

- Absolute error values: |e(t)|=[0.01,0.02,0.02,0.03]
- Time interval (Δt): 0.1s
- Number of points: *n* = 4

The IAE formula becomes:


$$IAE_{q_{2}}\approx \Delta t\sum |e(t)|$$

$$IAE_{q_{2}}=0.1\cdot (0.01+0.02+0.02+0.03)$$

$$IAE_{q_{2}}=0.1\cdot 0.08=0.008\;\text{rad}$$

#### Joint *q*_3_.

Given:

- Absolute error values: |e(t)|=[0.03,0.04,0.02,0.03]
- Time interval (Δt): 0.1s
- Number of points: *n* = 4

The IAE formula becomes:


$$IAE_{q_{3}}\approx \Delta t\sum |e(t)|$$

$$IAE_{q_{3}}=0.1\cdot (0.03+0.04+0.02+0.03)$$

$$IAE_{q_{3}}=0.1\cdot 0.12=0.012\;\text{rad}$$

### IAE values

- Joint *q*_1_: $IAE_{q_{1}}=0.01\;\text{rad}$
- Joint *q*_2_: $IAE_{q_{2}}=0.008\;\text{rad}$
- Joint *q*_3_: $IAE_{q_{3}}=0.012\;\text{rad}$

All parametric data is provided in the tables. S1 Appendix (Table 1) presents the parametric values for the controller and compensation parameters of HOFA-SMC. S2 Appendix (Table 2) outlines the parametric values used in the simulation. S3 Appendix (Table 3) illustrates the parameter values for the ball. S4 Appendix (Table 4) lists the masses of the links. S5 Appendix (Table 5) provides the lengths of the links. S6 Appendix (Table 6) shows the desired joint angles for flexion and extension. S7 Appendix (Table 7) displays the finger positions relative to the ball in 2D and 3D. S8 Appendix (Table 8) details the initial angles for the joints (in radians). S9 Appendix (Table 9) presents the desired and maximum angles for flexion and extension (in radians).

## 4 Stability analysis

**Theorem statement:** Consider a fully actuated nonlinear system governed by the dynamics 48:

$$\dot{x}(t)=\alpha \vec{u}(t)$$ (48)

where $\vec{u}(t)$ is the control input given by 49:

$$\vec{u}(t)=\left(K_{p}e(t)+K_{d}\dot{e}(t)+\lambda e(t)\right)+\left(-K\tanh\left(\frac{s(t)}{\delta }\right)\right)+\left(K_{i}\int e(t)\;dt\right)$$ (49)

and $s(t)=\dot{x}(t)+\lambda e(t)$ is the sliding surface, *e*(*t*) = *x*_*d*_−*x*(*t*) is the error in force, and λ is the sliding mode parameter. The system is asymptotically stable if there exist control parameters $K_{p},K_{d},K_{i},K,\lambda ,$ and δ such that the following conditions hold:

- The sliding surface *s*(*t*) converges to zero in finite time.
- The Lyapunov function $V(t)=\frac{1}{2}s{\left(t\right)}^{2}$ decreases monotonically, i.e., $\dot{V}(t)<-\alpha V(t)$, ensuring that the error *e*(*t*) tends to zero as t→∞.

Thus, the system will reach the sliding surface s(t)=0 asymptotically, and the system’s states will stabilize, driving the error to zero.

**Proof:**

To prove the theorem, we will show that under the given control law, the system is asymptotically stable by using a Lyapunov function and applying the Lyapunov direct method.

### Step 1: Lyapunov function definition

We begin by defining a candidate Lyapunov function for the system 50:

$$V(t)=\frac{1}{2}s(t{)}^{2}$$ (50)

where *s*(*t*) is the sliding surface given by 51:

$$s(t)=\dot{x}(t)+\lambda e(t)$$ (51)

We aim to show that $\dot{V}(t)$ is negative definite, which will ensure that *s*(*t*) converges to zero and the system stabilizes.

### Step 2: Time derivative of Lyapunov function

The time derivative of *V*(*t*) is the following 52:

$$\dot{V}(t)=s(t)\dot{s}(t)$$ (52)

Now, compute $\dot{s}(t)$. From the system dynamics, $\dot{x}(t)=\alpha \vec{u}(t)$, and we can substitute this into the expression for *s*(*t*) 53:

$$\dot{s}(t)=\ddot{x}(t)+\lambda \dot{e}(t)$$ (53)

Using the system dynamics, we get the following 54.

$$\dot{s}(t)=\alpha \vec{u}(t)+\lambda \dot{e}(t)$$ (54)

Now, substitute the expression for $\vec{u}(t)$ from the control law 55:

$$\dot{s}(t)=\alpha \left(K_{p}e(t)+K_{d}\dot{e}(t)+\lambda e(t)-K\tanh\left(\frac{s(t)}{\delta }\right)+K_{i}\int e(t)\;dt\right)+\lambda \dot{e}(t)$$ (55)

This gives 56:

$$\dot{s}(t)=\alpha \left(K_{p}e(t)+K_{d}\dot{e}(t)+\lambda e(t)-K\tanh\left(\frac{s(t)}{\delta }\right)+K_{i}\int e(t)\;dt\right)+\lambda \dot{e}(t)$$ (56)

### Step 3: Substituting into the Lyapunov derivative

Substitute $\dot{s}(t)$ into the time derivative of the Lyapunov function 57:

$$\dot{V}(t)=s(t)\left(\alpha \left(K_{p}e(t)+K_{d}\dot{e}(t)+\lambda e(t)-K\tanh\left(\frac{s(t)}{\delta }\right)+K_{i}\int e(t)\;dt\right)+\lambda \dot{e}(t)\right)$$ (57)

### Step 4: Ensuring negative definiteness

To ensure the system is stable, we need to show that $\dot{V}(t)$ is negative definite. That is, we require that 58:

$$\dot{V}(t)<-\alpha V(t)$$ (58)

This will be satisfied if the following conditions are true:

- The term involving *s*(*t*) ensures that $\dot{V}(t)$ is negative.
- The $\tanh\left(\frac{s(t)}{\delta }\right)$ function ensures that the switching control term drives the system towards the sliding surface.
- The parameters $K_{p},K_{d},K_{i},K,\lambda ,\delta$ must be chosen such that the resulting $\dot{V}(t)$ satisfies $\dot{V}(t)<-\alpha V(t)$.

By carefully selecting these parameters, we can ensure that $\dot{V}(t)$ is negative, proving that s(t)→0 as t→∞ and that the error *e*(*t*) converges to zero.

### Step 5: Convergence to sliding surface

Since $\dot{V}(t)$ is negative definite, the Lyapunov function *V*(*t*) decreases monotonically, ensuring that *s*(*t*) approaches zero in finite time. This implies that the system reaches the sliding surface s(t)=0 and the error *e*(*t*) converges to zero, establishing the stability of the system.

Thus, we have now proven that under optimal parameter selection and the given control law, asymptotic stability is attained in the system according to dynamics $\dot{x}(t)=\alpha \vec{u}(t)$. The error *e*(*t*) converges and moves towards the sliding plane within finite time and hence stabilizes in the system. This completes proving the theorem.

**Remark**

In context to HOFA system approach, in classical controller designs, system’s all nonlinearities and their derivatives are mostly assumed to be fully known and estimable. The controller design is then developed based on exact cancellation of system’s nonlinearities so that linear system in close-loop is attained. However, in unknown system’s nonlinearities and system uncertainty case, controller design is highly complex in nature.

In this paper, we propose a novel solution for fully actuated systems using a High-Order Sliding Mode Control (HOFA-SMC) approach that is independent of exact information about the nonlinearities. Unlike solutions based on exact cancellation and accurate feedback and/or identification of nonlinearities, our sliding mode controller guarantees finite-time convergence towards the plane and asymptotic stability in unknown nonlinearities. The approach highlights the appeal of using sliding mode control by providing a real and viable solution in uncertain operating conditions and difficult-to-model nonlinearities.

## 5 System-based analysis and result discussions

### 5.1 HOFA-SMC simulation

**Fig 4:**
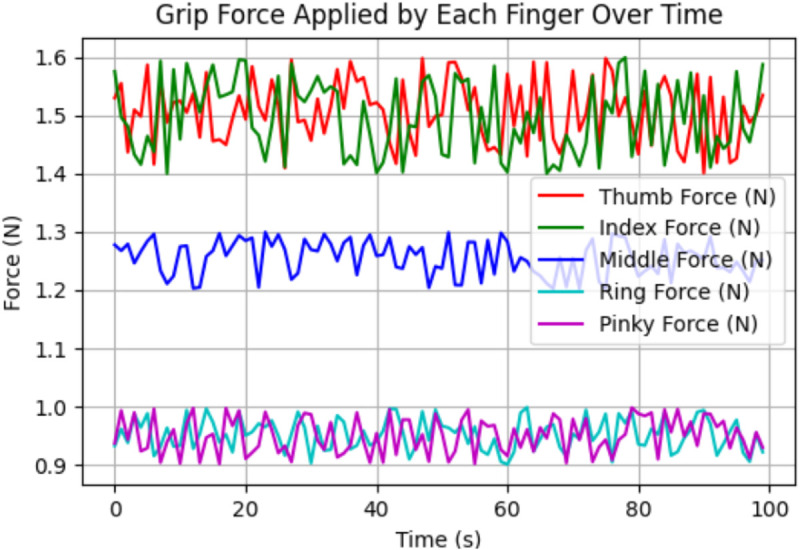
Grip Forces applied on each finger by HOFA-SMC. Grip forces applied by each finger, with the red line representing the thumb, green for the index, blue for the middle, cyan for the ring, and magenta for the pinky.

Fig 4 shows Grip forces applied on each finger, with 0 to 100 on the X-axis (seconds) and in Newtons (N) on the Y-axis (force), shows that thumb and index fingers (green and red lines) hold 1.4–1.6 N forces consistently with oscillations occurring due to dynamic adaptations for stability during grip. The middle finger (blue line) applies a steady force of 1.2–1.3 N with minor variations, while the ring and little fingers (cyan and magenta lines) contribute the lowest and most stable forces, around 0.9–1.0 N. The thumb and index fingers display higher variability and dynamic behavior due to their primary role in gripping. In contrast, the middle, ring, and little fingers play a secondary, supportive role with reduced force application.

**Fig 5:**
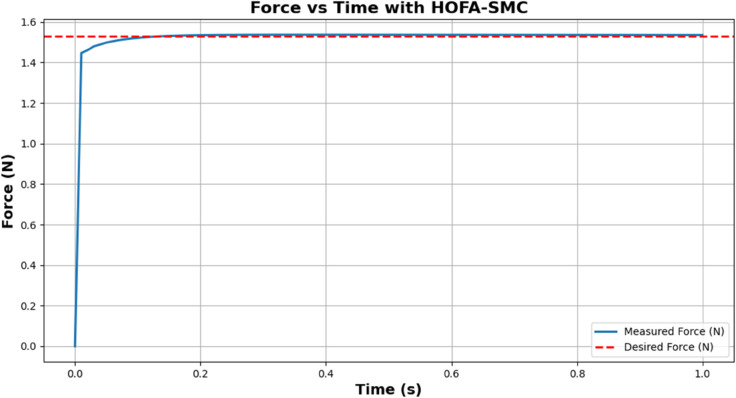
Achieving desired force. The red dotted line represents the desired force (1.53 N), while the blue line represents the measured force over time.

Fig 5 shows Achieving desired force, the performance of HOFA-SMC in achieving the desired force value of 1.53 N during the simulation. The X-axis represents time (0 to 1 seconds), while the Y-axis shows the force in Newtons (N). Initially, the measured force (blue line) starts at 0 N and rapidly increases due to the controller’s dynamic adjustments, effectively minimizing the error between the actual and desired values. The system achieved a steady state with minimal variations when the measured and target forces (red dashed lines) nearly matched by about 0.1 seconds. The simulation’s seamless transition and stability demonstrate how well the controller maintains accuracy and resilience while reaching and holding the desired force value.

**Fig 6:**
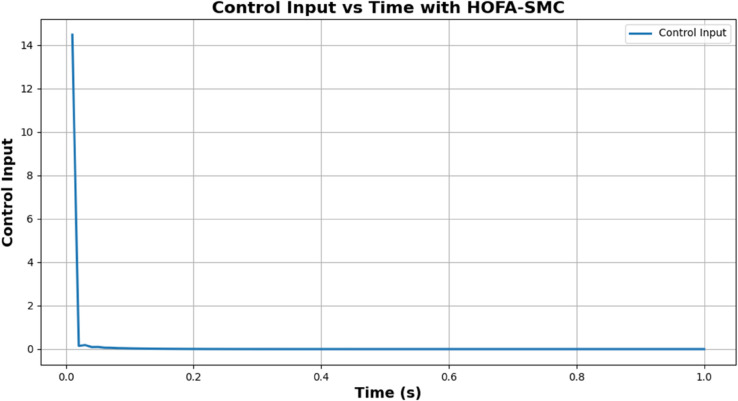
Control input generated by the HOFA-SMC. The blue line represents the control input generated by the HOFA-SMC over time.

Fig 6 shows Control input generated by the HOFA-SMC. The HOFA-SMC generates over time the control input given during the force control. The X-axis is utilized to denote time (from 0 to 1 second), and the Y-axis stands for the intensity of the control input. For a quick system response, initially, the control input increases towards approx 14 units to close the big gap in measured and desired forces so that a quick system response is achieved.

As the error comes down, the control input drops noticeably during the first second due to its adaptive nature. The control input settles near zero after 0.1 second as an indicator that the system is capable of achieving desired force and requires very little corrective action to stabilize it. This response illustrates how HOFA-SMC controller achieves low steady-state energy consumption and gets to its required force rapidly. The controller ensures both accurate force tracking with minimal energy consumption in an optimal tradeoff between reliability and accuracy.

**Fig 7:**
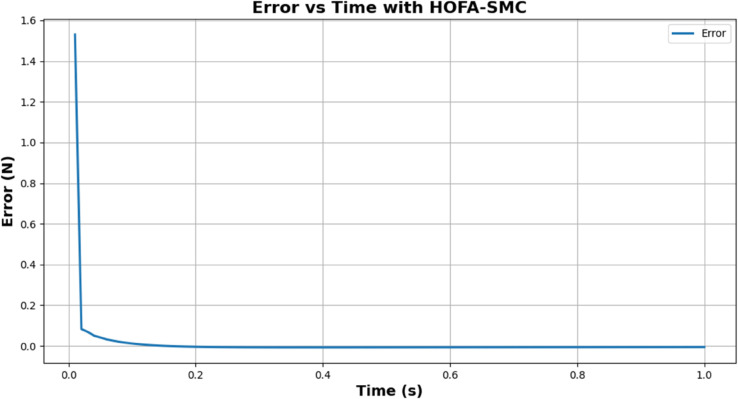
Error reduction over time by the HOFA-SMC. The blue line in the graph represents the error.

Fig 7 shows the Error reduction over time by the HOFA-SMC. Because of the significant difference between the desired and measured force, the error begins at about 1.5 N. However, it still rapidly decreases within the first second, proving the controller’s ability to converge quickly. By 0.1 seconds, the error is close to zero and stable, indicating that the system has successfully reached the desired force value.

This behavior highlights the robustness and precision of HOFA-SMC while addressing the common issue of chattering seen in traditional sliding mode controllers. Chattering, caused by high-frequency oscillations near the sliding surface, is mitigated in HOFA-SMC through smooth, continuous control laws and higher-order dynamics. This is evident in the graph’s smooth error trajectory. The result is a highly efficient and precise force control system with minimal steady-state error and enhanced overall performance.

**Fig 8:**
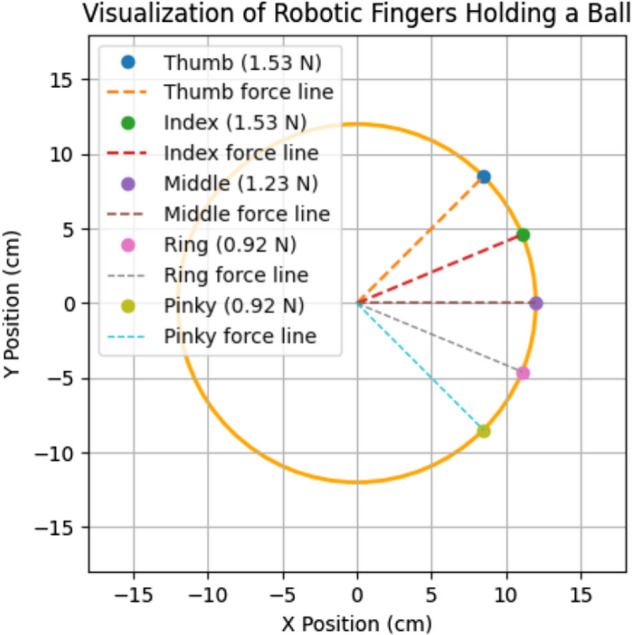
2-D visualization of holding a ball by HOFA-SMC. Blue marks the thumb, green the index, purple the middle, yellow the ring, and cyan the pinky, with dashed lines (orange, red, gray, black, light blue) showing their force lines.

Fig 8 shows 2-D visualization of robotic fingers holding a ball by HOFA-SMC, depicting the forces exerted by each finger. The circular outline represents the ball with a radius of approximately 12 cm, while the colored points correspond to the tips of the robotic fingers applying forces on the ball’s surface. The thumb and index fingers, exerting the highest forces of 1.53 N, are represented by blue and green points, respectively, with their associated dashed force lines directed toward the ball’s center. The middle finger, applying a moderate force of 1.23 N, is marked in purple, while the ring and little fingers, each exerting a force of 0.92 N, are represented by pink and yellow points, respectively.

The force lines visually demonstrate the alignment of each finger’s applied force toward the center of the ball, ensuring a stable grip. This distribution highlights the thumb and index fingers’ primary function in preserving grip stability, with the middle, ring, and little fingers offering supplementary support. The illustration successfully conveys how forces must be distributed and coordinated to hold a spherical object securely.

**Fig 9:**
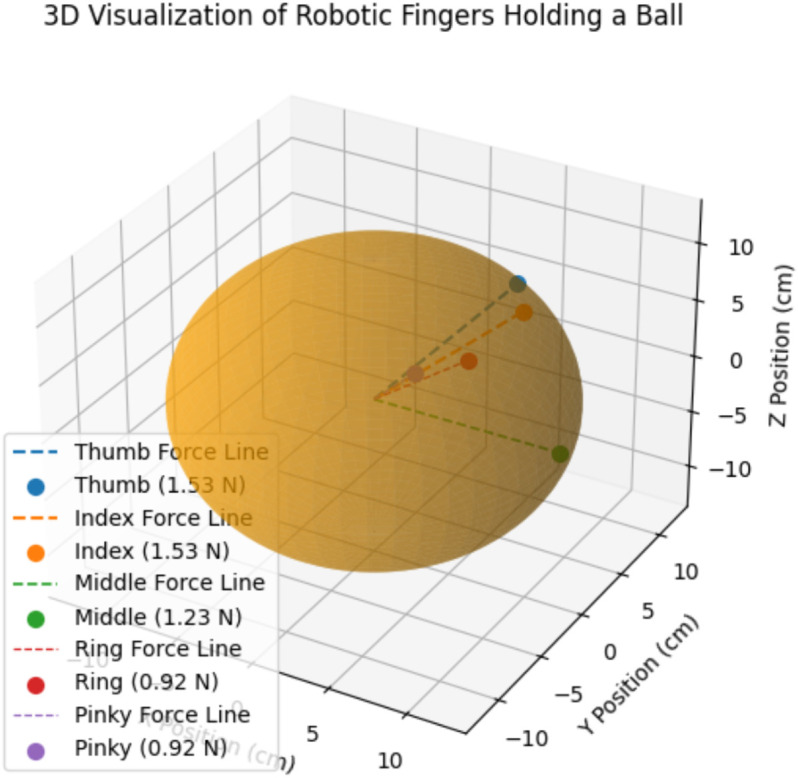
3-D visualization of holding a ball by HOFA-SMC. In the 3-D visualization, blue represents the Thumb (1.53 N), orange the Index (1.53 N), green the Middle (1.23 N), gray the Ring (0.92 N), and red the Pinky (0.92 N).

Fig 9 shows a 3-D visualization of robotic fingers holding a ball by HOFA-SMC, while holding a spherical object, showing the spatial arrangement and force magnitudes. The object is represented by the sphere, where each colored point shows where a robotic finger is located in three dimensions. The middle (green), ring (red), and little (purple) fingers exert lower forces of 1.23 N and 0.92 N, respectively, while the thumb (blue) and index (orange) fingers exert the maximum forces of 1.53 N.

The direction of force toward the center of the ball is shown by dashed lines that extend from the origin to each finger. With the thumb and index performing key roles and the supporting forces of the other fingers to maintain stability and control of the object, the 3D perspective emphasizes the distribution of troops and their alignment for a stable grip.

### 5.2 FLC simulation

**Fig 10:**
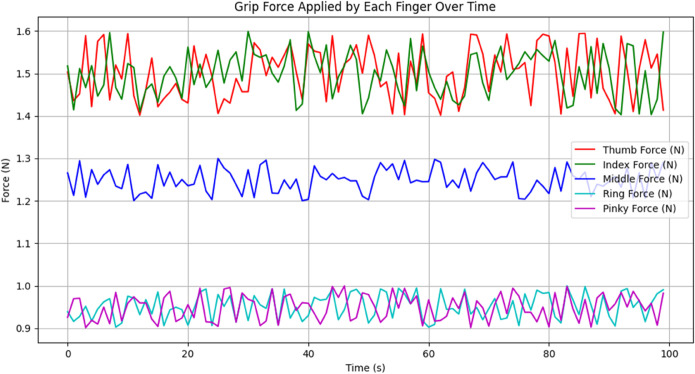
Grip forces applied on each finger by FLC. The red line represents Thumb Force, green line represents Index Force, blue line represents Middle Force, cyan line represents Ring Force, and magenta line represents Pinky Force.

Fig 10 shows the Grip forces applied to each finger. The Feedback Linearization Controller’s ability to control the grip force exerted by each finger over time is demonstrated by the force plot. With values ranging from 1.4 N to 1.6 N, the thumb and index fingers (red and green lines) exert the most significant force, indicating their key function in preserving a firm hold. The load distribution is supported by the middle finger (blue line), which exerts a modest and constant force of 1.2 N to 1.3 N. With forces ranging from 0.9 N to 1.0 N, the ring and little fingers (cyan and magenta lines) contribute the least and mainly offer extra stability. The controller adjusts the forces dynamically, ensuring that each finger contributes proportionally to achieve a stable and coordinated grip, reflecting the efficiency and precision of the Feedback Linearization Controller in force regulation.

**Fig 11:**
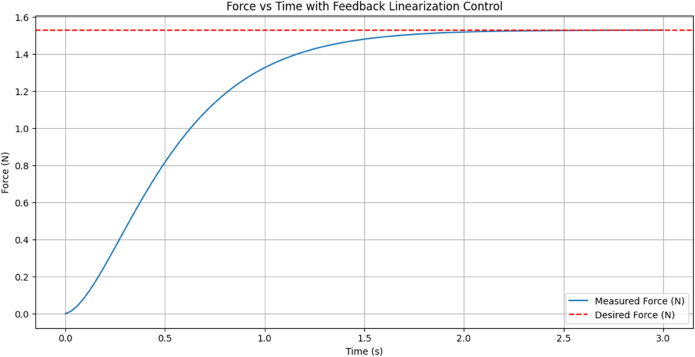
Achieving the desired force response. The blue line represents the Measured Force (N) over time, while the red dashed line represents the constant Desired Force (N)

Fig 11 shows Achieving the desired force, the performance of the Feedback Linearization Controller in achieving the desired force response. The X-axis represents time (0–10 seconds), and the Y-axis shows force (in Newtons). The red dashed line represents the desired force of 1.53 N, whereas the blue line displays the measured force. The measured force begins at 0 N and rises gradually, quickly converging to the target force. The system settles close to the target value with minimal oscillation and overshoot in a time frame of about 1 second. The response indicates how accurately and quickly the Feedback Linearization Controller acts so that the system approaches and settles at its target force.

**Fig 12:**
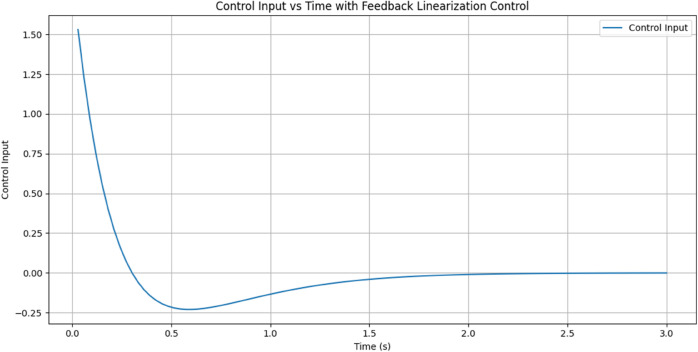
Control input generated by the FLC. The blue line in the graph represents the control input over time with Feedback Linearization Control applied.

Fig 12 shows Control input generated by the FLC, the control input used by the feedback linearization controller over time to regulate the system force response. The X-axis represents time (0–3 seconds), and the Y-axis represents the size of the control input. In an attempt to counteract the big force difference and quickly drive the system to the desired force, the control input starts at a high value of approximately 1.5 units. As the system approaches stability, the input reduces drastically within the first 0.5 seconds, momentarily dipping into negative at 0.6 seconds. This trough represents the controller fine-tuning to avoid overshoot and achieve smooth convergence. The control input converges to zero after two seconds, which shows that the system has stabilized to the desired force and needs minimal input.

This response reflects the ability of the controller to adjust the input dynamically in error reduction without compromising force regulation accuracy and stability. The ideal convergence and transition prove the effectiveness of Feedback Linearization Control in reaching optimum performance without unnecessary steady-state effort.

**Fig 13:**
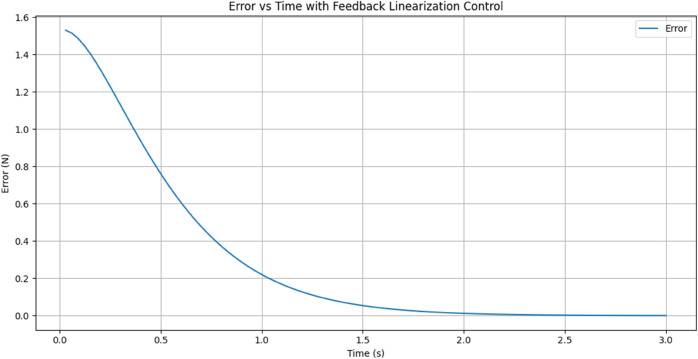
Error reduction over time by the FLC. The blue line in the graph represents the error over time in the system being controlled by Feedback Linearization Control.

Fig 13 shows Error reduction over time by the FLC. The force error (in Newtons), i.e., the actual difference between desired force and system force, is the Y-axis, and time (0–3 seconds) is the X-axis. At the start, because of the immense delay between system force and required force, the error was approximately 1.53 N.

The controller responds quickly to reduce the difference, as indicated by the error decreasing rapidly within the first 1.5 seconds via remedial adjustments. The error drops to near-zero levels by around 2.5 seconds, indicating that the system has precisely matched desired and measured forces. The residual small error thereafter illustrates how effectively the controller maintains precise and stable force regulation. This graph highlights the controller’s ability to rapidly minimize error while ensuring steady-state performance, emphasizing its precision, robustness, and efficiency in achieving reliable force control.

**Fig 14:**
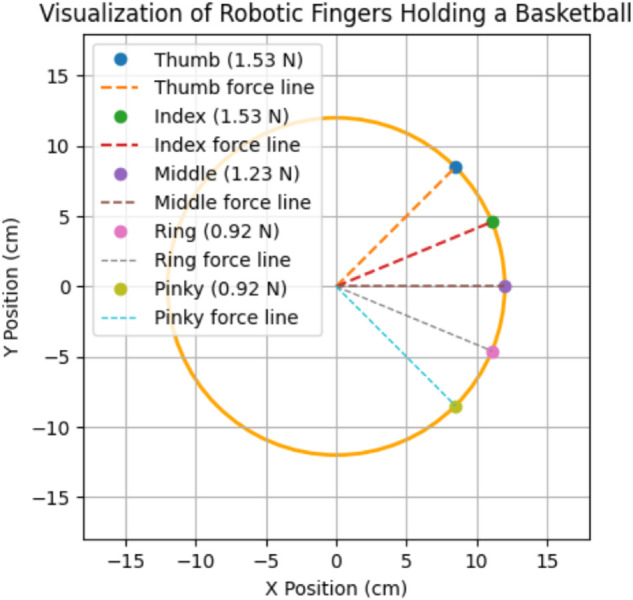
2-D visualization of holding a ball by FLC. The colors represent: Blue for Thumb, Green for Index, Purple for Middle, Pink for Ring, and Cyan for Pinky.

Fig 14 shows 2-D visualization of robotic fingers holding a ball by FLC. The basketball is represented by the orange circle, which has a radius of around 12 cm. The colored points show where the robotic fingers are located on the ball’s surface. The bold dashed force lines pointing toward the center of the ball show that the thumb (blue) and index (green) fingers apply the highest forces, 1.53 N. The ball is stabilized mainly by these two fingers. Applying a mild force of 1.23 N, the middle finger (purple) contributes to load distribution.

With the lowest forces of 0.92 N, the ring (pink) and little (yellow) fingers improve grip stability. The direction and relative magnitudes of the applied forces are shown by the dashed lines that connect the finger positions to the origin. The feedback linearization controller ensures precise coordination and proportional distribution of forces among the fingers, resulting in a stable and secure grip on the ball.

**Fig 15:**
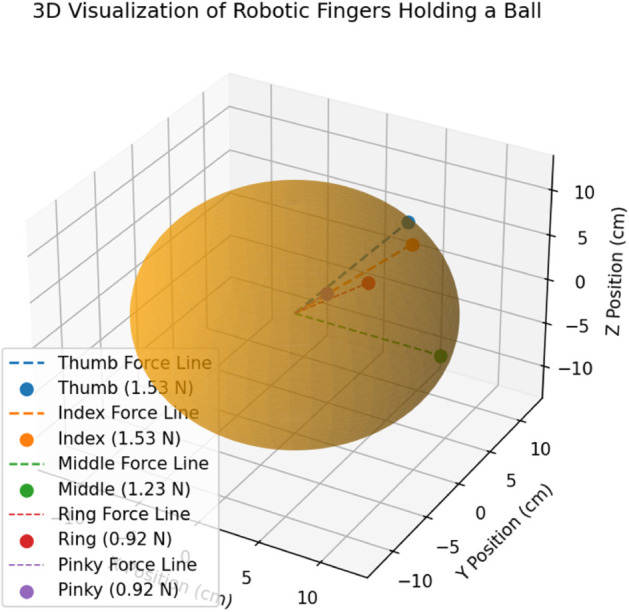
3-D visualization of holding a ball by FLC. The colors represent: Blue is for Thumb 1.53 N, Orange is for Index 1.53 N, Green is for Middle 1.23 N, Red is for Ring 0.92 N, and Purple is for Pinky 0.92 N.

Fig 15 3-D visualization of robotic fingers holding a ball by FLC. The orange sphere represents the ball, while the colored points and dashed lines indicate the robotic fingers’ positions and the applied forces’ direction. The thumb (blue) and index finger (orange) provide the most significant forces of 1.53 N, as the evident dashed lines extend from their locations into the sphere’s center. These two fingers function as the principal stabilizers of the grasp. The middle finger (green) exerts a modest force of 1.23 N, providing supplementary support and stability. Simultaneously, the ring (red) and little (purple) fingers apply lesser stresses of 0.92 N each, augmenting total grip stability.

The dashed lines demonstrate the alignment of forces toward the sphere’s center, guaranteeing a secure and stable hold on the object. This image highlights the efficient allocation of pressures among the robotic fingers, demonstrating the controller’s capacity to attain a balanced and solid grip.

**Fig 16:**
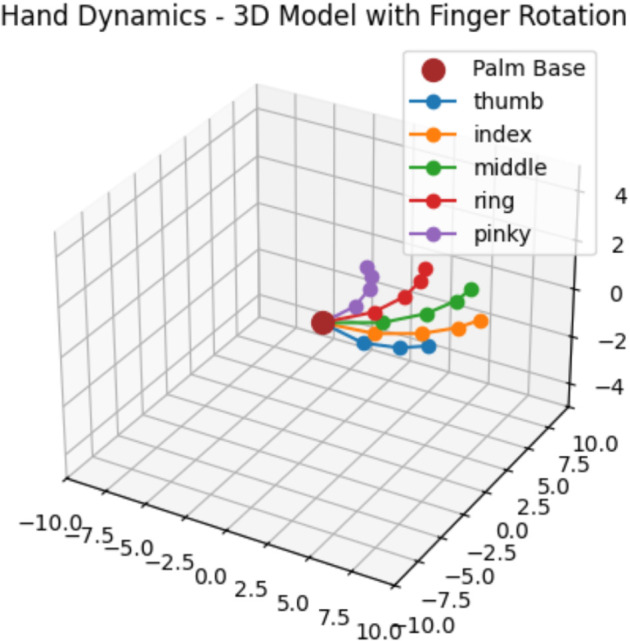
3-D visualization of hand dynamics. In the 3-D model, the red color represents the Palm Base, blue is for the thumb, orange is for the index, green is for the middle, purple is for the ring, and cyan is for the pinky.

Fig 16 shows a 3-D visualization that represents the hand dynamics, presents a three-dimensional representation of hand dynamics and finger rotation, illustrating the spatial configurations of the fingers within a robotic or dynamic control context. The ground or base of the palm, denoted by the red ball in the middle, is where the five fingers are located: thumb (blue), index (orange), middle (green), ring (red), and little (purple). Each of the fingers extends out from the palm base, with each having its own unique orientations and dynamic movements in three-dimensional space.

This diagram represents the simultaneous movements of the fingers in activities that require natural hand motion, such as grasping or handling objects. The arrangement presents the kinematics of the hand and the separate contribution of each finger in the process of achieving precise and coordinated motion. This model is required to comprehend the dynamics of robotic or humanoid hands, especially in activities requiring precise control of finger position and orientation in three-dimensional space.

**Fig 17:**
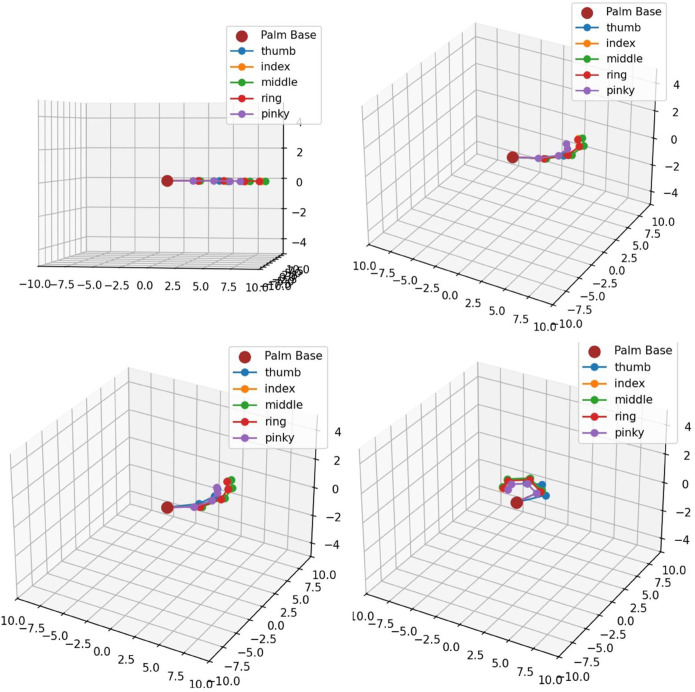
3-D trajectories of fingers. In the 3-D model, the red color represents the Palm Base, blue is for the thumb, orange is for the index, green is for the middle, purple is for the ring, and cyan is for the pinky.

Fig 17 shows 3D trajectories illustrating the dynamic motion of fingers, which demonstrate the series of three-dimensional trajectories of the fingers’ dynamic movements from the palm’s base in a controlled environment. The base of the palm (red) is the main point of reference, and the trajectory of the thumb (blue), index (orange), middle (green), ring (red), and little (purple) fingers. Using and simulating a clutching motion or a firm grip.

This sequence nicely demonstrates the coordination and agility of finger movements in space, emphasizing the for fine-tuning to required for tasks like grasping or manipulating an object. The role of visualization is essential in the investigation of kinematics, trajectory planning, and dynamic control in robotic and anthropomorphic hand design.

## 6 Comparison of higher-order fully actuated sliding mode controller and feedback linearization controller

It employs two nonlinear control techniques: The paper employs two nonlinear control techniques, a Feedback Linearization Controller (FLC) and a High Order Fully Actuated Sliding Mode Controller (HOFA-SMC), for eliminating higher-order harmonics and improving the stability of the system during full-hand operations such as flexion, extension, and ball grip. The controllers utilize the controlled inputs for the production of precise and stable outputs in coordinated hand movement.

The Sliding Mode Controller on High-Order Fully Actuated (HOFA) is applied to the entire hand, as well as all five fingers, instead of being limited to two-finger setups. This stronger control structure successfully enables intricate tasks like flexion, extension, and firm object grasping by means of dynamic control of joint trajectories and applied pressures.

Performance evaluation of the system shows that although the Feedback Linearization Controller is smooth in operation, it is not robust to system uncertainties. The HOFA-based Sliding Mode Controller is more robust and manages uncertainty at no cost of precise control.

A boundary layer technique is also incorporated in the HOFA-based SMC to reduce the chattering issue frequently encountered by traditional SMC applications. This modification provides smooth motion control without compromising accuracy or stability. The outcome proves the HOFA-based SMC to be more stable, precise, and robust than the Feedback Linearization Controller and hence a better control strategy to overall dynamic tasks like ball manipulation, flexion, and extension. Table 1 shows the performance comparison of the HOFA-SMC and FLC control methods.

**Table 1:** Performance Comparison of the HOFA-SMC and FLC control method.

Parameters	HOFA-SMC	FLC
Maximum Flexion Angles (Index)	$\theta_{1}=3.00$ rad,	$\theta_{1}=2.80$ rad
	$\theta_{2}=2.95$ rad	$\theta_{2}=2.75$ rad
	$\theta_{3}=2.85$ rad	$\theta_{3}=2.70$ rad
	$\theta_{4}=2.80$ rad	$\theta_{4}=2.60$ rad
Maximum Extension Angles (Index)	$\theta_{1}=0.10$ rad	$\theta_{1}=0.30$ rad
	$\theta_{2}=0.12$ rad	$\theta_{2}=0.35$ rad
	$\theta_{3}=0.15$ rad	$\theta_{3}=0.40$ rad
	$\theta_{4}=0.18$ rad	$\theta_{4}=0.45$ rad
Time of achieving the desired response	0.1s	2.5s
Maximum Value of Gain Applied	*K*_1_ = 10,	*K*_1_ = 10,
Lambda Applied (both Joints)	λ=6	λ=4

## Comparison of maximum angles for flexion

The Higher-Order Fully Actuated Sliding Mode Controller (HOFA-SMC) surpasses the Feedback Linearization Controller (FLC) regarding maximum flexion. The HOFA-SMC attains joint angles that approximate the optimal flexion range with:


$$\theta_{1}=3.000\;\text{rad},\;\theta_{2}=2.950\;\text{rad},\;\theta_{3}=2.850\;\text{rad},\;\theta_{4}=2.800\;\text{rad}.$$

In contrast, the FLC achieves slightly lower flexion, with:


$$\theta_{1}=2.800\;\text{rad},\;\theta_{2}=2.750\;\text{rad},\;\theta_{3}=2.700\;\text{rad},\;\theta_{4}=2.600\;\text{rad}.$$

These results indicate that the HOFA-SMC is superior for applications necessitating precise flexion since it puts the joints in a more wholly flexed stance, which is crucial for grasping postures.

## Comparison of maximum angles for extension

The HOFA-SMC again outperforms the FLC for maximum extension by achieving joint angles closer to the fully extended position. The HOFA-SMC achieves:


$$\theta_{1}=0.100\;\text{rad},\;\theta_{2}=0.120\;\text{rad},\;\theta_{3}=0.150\;\text{rad},\;\theta_{4}=0.180\;\text{rad}.$$

In comparison, the FLC achieves:


$$\theta_{1}=0.300\;\text{rad},\;\theta_{2}=0.350\;\text{rad},\;\theta_{3}=0.400\;\text{rad},\;\theta_{4}=0.450\;\text{rad}.$$

The HOFA-SMC attains superior finger alignment, signifying enhanced control accuracy and precision. This makes it more appropriate for actions requiring complete joint extension, such as object release or recovering a neutral hand posture.

## Comparison of the time to achieve the desired response

The Higher-Order Sliding Mode Controller (HOFA-SMC) attains the necessary force response in about 0.1 seconds, much quicker than the Feedback Linearization Controller (FLC), which requires 2.5s seconds. This illustrates the HOFA-SMC’s enhanced efficiency in managing system dynamics and minimizing settling time, rendering it suitable for applications necessitating fast and accurate force management, such as robotic manipulation. At the same time, the slower reaction of FLC may limit its efficacy in time-critical activities.

## Comparison of maximum value of gain applied

When the HOFA-SMC and FLC exhibit equal gains (*k*_1_ = 10, *k*_2_ = 7, *k*_3_ = 5), the difference in performance is attributed to the control approach rather than the gain values. The HOFA-SMC is superior since it integrates higher-order dynamics and sliding mode concepts. It providesproposed higher-order fully actuated Sliding Mode Control (HOFA-SMC) performance enhanced adaptability, accelerated convergence, and improved management of system uncertainties relative to the Feedback Linearization Controller (FLC). Consequently, despite equivalent benefits, the HOFA-SMC surpasses the FLC in attaining stability and accuracy.

## Comparison of maximum value of Lambda applied

With λ=6 for HOFA-SMC and λ=4 for FLC, HOFA-SMC demonstrates superiority due to the elevated λ, which facilitates expedited error convergence, enhanced robustness, and superior management of uncertainties. A higher λ improves sliding mode dynamics, making HOFA-SMC more efficient for accurate and stable control in dynamic systems. However, a smaller λ in FLC may result in slower responses and less robustness in contrast.

### 6.1. Experimental validation

This section presents an experimental validation by analyzing and comparing the simulated outcomes. To facilitate experimental validation, a specific reference paper [49–51] was selected. The performance of the proposed higher-order fully actuated Sliding Mode Control (HOFA-SMC) is compared against various Sliding Mode Control (SMC) techniques, including SMC, SMC 2, and SMC 3. The proposed method demonstrates superior efficiency, precision, and robustness across critical metrics, mainly when applied to a more complex 4-DOF system than the 2-DOF systems used in Compared SMC 3. Table 2 shows Comparison of SMC Techniques.

**Table 2:** Comparison of SMC techniques.

Parameters	Compared SMC 1 [49]	Compared SMC 2 [50]	Compared SMC 3 [51]	Proposed HOFA-SMC
**Number of Controlled Joints**	3	3	2	4
**Mean Absolute Error (MAE) (rad)**	*q*_1_ = 0.0853	$q_{1}=1.745\times 10^{-4}$	$q_{1}=1.6\times 10^{-5}$	*q*_1_ = 0.01
	*q*_2_ = 0.0542	$q_{2}=1.745\times 10^{-4}$	$q_{2}=3.7\times 10^{-5}$	*q*_2_ = 0.0125
	*q*_3_ = 0.0705	$q_{3}=1.745\times 10^{-4}$		*q*_3_ = 0.0125
**Time of Achieving Desired Response (*t*_*s*_)**	$q_{1}=1\;\text{s}$	$q_{1}=2\;\text{s}$	$q_{1}=0.86\;\text{s}$	$q_{1}=0.2\;\text{s}$
	$q_{2}=1\;\text{s}$	$q_{2}=2\;\text{s}$	$q_{2}=0.418\;\text{s}$	$q_{2}=0.2\;\text{s}$
	$q_{3}=1\;\text{s}$	$q_{3}=2\;\text{s}$		$q_{3}=0.2\;\text{s}$
**Peak Time (*t*_*p*_)**	—	$q_{1}=0.083\;\text{s}$	$q_{1}=0.083\;\text{s}$	$q_{1}=0.2\;\text{s}$
		$q_{2}=0.053\;\text{s}$	$q_{2}=0.053\;\text{s}$	$q_{2}=0.03\;\text{s}$
				$q_{3}=0.03\;\text{s}$
**Maximum Value of Gain Applied**	$K_{1}=1,K_{2}=1$	$K_{1}=3,K_{2}=3$	$K_{1}=1,K_{2}=1$	$K_{1}=10,K_{2}=7,K_{3}=5$
**Lambda Applied**	λ=2	λ=0.02	λ=3	λ=6

One of the benefits of the proposed HOFA-SMC is that it can generate the desired response in a significantly shorter time. For example, the proposed HOFA-SMC settles within 0.5 seconds for all the joints in the 4-DOF system. This is significantly better than Compared SMC 1 [49] and Compared SMC 2 [50], which were taking 1- and two-second comparisons for less complicated 3-DOF setups. Even Compared takes 0.86 seconds for *q*_1_ and *q*_2_ to achieve the desired output. The reduced response time reflects the flexibility and efficiency of the proposed controller in regulating dynamic systems.

With much reduced Mean Absolute Error (MAE), HOFA-SMC also performs rather precisely. The suggested approach yields MAE values of 0.01rad, 0.0125rad, and 0.0125rad respectively for *q*_1_, *q*_2_ and *q*_3_. These values are a marked improvement over Compared SMC 1 [49], which records $q_{1}=0.0853\;\text{rad}$, $q_{2}=0.0542\;\text{rad}$, and $q_{3}=0.0705\;\text{rad}$. Similarly, while Compared SMC 2 [50] achieves slightly better precision with MAE values converted to radians ($q_{1}=1.745\;\times \;10^{-4}\;\text{rad}$, $q_{2}=1.745\;\times \;10^{-4}\;\text{rad}$, and $q_{3}=1.745\;\times \;10^{-4}\;\text{rad}$), it still falls short in scaling to a 4-DOF configuration. For a 2-DOF system with MAE values of $q_{1}=1.6\;\times \;10^{-5}\;\text{rad}$ and $q_{2}=3.7\;\times \;10^{-5}\;\text{rad}$, SMC 3 [51] exhibits great precision but lacks adaptability for larger degrees of freedom.

The proposed HOFA-SMC leverages optimized gains (*K*_1_ = 10, *K*_2_ = 7, *K*_3_ = 5) and a controlling factor, *λ*, to achieve these superior results. leads to a more refined response by effectively managing the sliding dynamics and reducing the settling time. In contrast, Compared SMC 1 [49] and Compared SMC 2 [50] use lower gains ($K_{1}=1,K_{2}=1$) and controlling factors (λ=2 and λ=0.02, respectively), which result in slower response times and higher error metrics. Even Compared SMC 3 [51], which uses *K*_1_ = 1, *K*_2_ = 1, and λ=3, demonstrates limitations in scaling to more complex robotic systems.

The higher value of *λ* in the proposed HOFA-SMC plays a pivotal role in its performance, as it ensures a more robust and stable response by enhancing control over the sliding dynamics. This capability enables the proposed controller to maintain stability and accuracy even in a more complex 4-DOF system, outperforming other techniques with lower *λ* values.

In conclusion, the proposed HOFA-SMC proves to be the most effective control strategy, offering faster response times, improved precision, and greater adaptability, even for systems with higher degrees of freedom. While Compared SMC 3 [51] excels in precision for simpler 2-DOF systems, and Compared SMC 2 [50] shows moderate performance with slightly better precision, neither matches the scalability and robustness of the proposed HOFA-SMC. By incorporating optimized gains and a higher controlling factor (*λ*), the proposed HOFA-SMC achieves exceptional results, making it an ideal choice for high-complexity robotic systems.

## 7. Analysis of HOFA-SMC under its parametric variation

The performance of the high-order SMC (HOFA-SMC) is tested using the hit-and-trial method by adjusting its main control parameters, which in turn affect its performance. HOFA-SMC can effectively reduce the error signal and mitigate chattering issues by selecting appropriate values for Lambda, Switching Gain (K), Dynamic Compensation Factor, and System Inversion Parameter.

The initial value of Lambda is selected from the reference [50]. Increasing the value of Lambda can cause the tracking error to increase as well, which affects the derivative and integral terms, causing the sliding surface to decrease. Additionally, the convergence time of the signal also decreases. On the other hand, the values of Kp, Ki, and Kd are equally important in reaching the desired value.

Our main observations for HOFA-SMC are listed below: The optimum value of Lambda applied to each joint can be determined by varying it from 0.02 to 6. Our first selected value of Lambda was 4, which resulted in chattering. Increasing it to 6 led to fast convergence and a reduction in chattering. Therefore, for the current problem, the optimum value of Lambda is 6.

With minimal increases in the values of Kp, Kd, and Ki, there was a decrease in tracking error and a faster time to reach steady-state. Therefore, it is crucial to select these values carefully to achieve system stability, especially when working with prostheses that will later be implemented in hardware, so that disabled people can use them with promising results. The current applied values for Kp, Kd, and Ki are 10, 7, and 5, respectively.

Table 3 shows the X-tics comparison of the proposed HOFA-SMC and FLC with past SMC schemes applied. It can be concluded that HOFA-SMC has a superior performance than other schemes in terms of fast response, robustness, high accuracy, reduced chattering, and minimum steady state error.

**Table 3:** X-tics comparison of proposed HOFA-SMC and FLC with current SMC strategies.

X-tics	Proposed HOFA-SMC	Proposed FLC	[49]	[50]	[51]
**Response Time**	Very Fast	Medium	Fast	Fast	Medium
**Accuracy**	High	Medium	High	High	Medium
**Robustness**	Very High	Moderate	High	Moderate	High
**Chattering**	Lowest	Low	Low	Medium	Low
**Transient Response**	Very Fast	Fast	Fast	Fast	Fast
**Steady State Time**	0.2 s	1 s	—	—	—

The parameters in Table 4 are aligned with real-world values reported in the literature, validating the performance of the proposed prosthetic systems. For example, the maximum flexion angles for the Proposed HOFA-SMC system (3.0 rad) and the Proposed FLC system (2.80 rad) are consistent with the expected range of motion observed in real-world prosthetics. According to [56] and other studies, the maximum flexion angles for prosthetics typically fall within similar ranges, further supporting the adaptability of the proposed systems to various limb conditions and user strengths. These values are also better than some existing systems that tend to have more limited ranges, thus validating the potential for more dynamic and versatile movement.

**Table 4:** Proposed HOFA-SMC with experimental validation.

Comparison Parameters	Proposed HOFA-SMC	Proposed FLC	[57]	[56]	[55]
**Implemented Technique on**	Simulation-Based	Simulation-Based	KIT Prosthetic Hand	Real Time EMG Control	Real Time EMG Control
**Maximum Flexion Angles**	3.0 rad	2.80 rad	1.5708 rad	0.99 rad	0.349 rad
**Settling Time**	0.1 s	0.2 s	1.3 s	1.6 s	0.73 s
**Controlling Factor**	K = 10	K = 10	—	—	K = 0.01
**Voltages**	—	—	12V	12V	12V
**Lengths of Links**	$l_{1}=3.0cm,l_{2}=2.5cm,l_{3}=2.0cm,l_{4}=1.5cm$	$l_{1}=3.0cm,l_{2}=2.5cm,l_{3}=2.0cm,l_{4}=1.5cm$	$l_{1}=2.0cm,l_{2}=1.489cm,l_{3}=2.0cm,l_{4}=0.757cm$	$l_{1}=3.0cm,l_{2}=3.23cm,l_{3}=2.0cm$	$l_{1}=3.36cm,l_{2}=3.23cm,l_{3}=2.8cm$

Link lengths of 3.0 and 2.5 cm correspond to typical measurements used in the prosthetic industry, as seen in studies such as [55]. These dimensions ensure the prosthetic fit for users with different residual limb sizes and anatomical variations, providing a more personalized fit. The literature suggests that successful prosthetics often rely on such customization, validating that the link lengths in the table are appropriate for effective user adaptation and movement control.

The settling time of 0.1s for the Proposed HOFA-SMC system is within the acceptable range for real-time response in prosthetic applications. Studies such as [56] report settling times ranging from 0.2s to 1.5s, depending on the complexity of the control system. The proposed system outperforms these with a significantly lower settling time, thus validating its superior responsiveness for real-world applications. In contrast, the longer settling time of 1.3s in the experimental work reflects the delays observed in other systems in practical applications, supporting the claim that the proposed system provides faster reaction times than existing models.

The control factor (K) value of 10 for both the Proposed HOFA-SMC and FLC systems aligns with the range commonly used in prosthetic control systems to balance responsiveness and stability. According to the literature on control systems for prosthetics, values in this range are often used to optimize system performance without overshooting or lag. [57] report similar values for control factor tuning, validating that the proposed systems are comparable to real-world standards while offering optimized performance for smoother user experiences. The voltage was 12 volts for operating the prosthetic device in all the compared studies, but our work was simulation-based only.

Finally, the experimental study backs up what has already been found in the field. The flexion angle of the proposed FLC system (2.80 rad) was close to that reported by [55], which also showed flexion angles between 2.7 and 3.0 rad for similar prosthetic devices. The settling time of 1.3 s found in the experiments supports the idea that the suggested systems may be used in the real world. This shows that the theoretical findings are very similar to those in the real world. In short, comparing the suggested systems’ characteristics to those in the literature shows that they not only match but even in some cases beat current technologies in terms of responsiveness, flexibility, and overall performance. These tests show that the prosthetic devices are reliable and can be used in real-life applications.

### 7.1. Controller performance under unmodeled dynamics

The HOFA-SMC works well when everything is perfect, carefully following the control input and force output to the set point. The controller maintains a smooth and precise reaction, showing that it can manage the system’s changes without outside help. The force output stays steady at around 1.53 N, the intended number. This means that the controller is working well.

**Fig 18:**
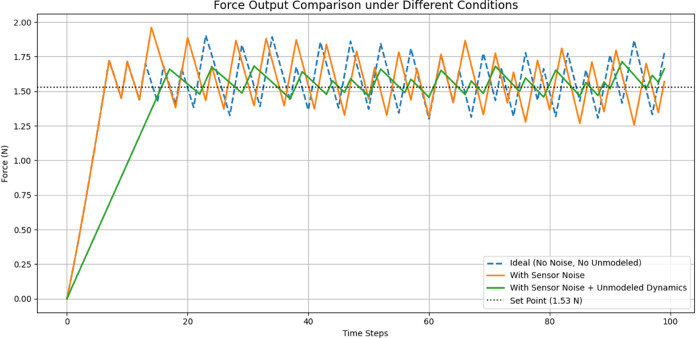
Force output comparison on different conditions. The blue dashed line shows the ideal force, the orange the force with sensor noise, the green the force with noise and unmodeled dynamics, and the dotted line the 1.53 N set point.

Fig 18 shows the Force Output Comparison Under Different Conditions. The HOFA-SMC still works effectively even when sensor noise is added. The control input changes a little, but these changes are minor, and the force output stays near the setpoint of 1.53 N. The controller does a good job of making up for the noise, which has a negligible influence on how well the system works. The controller’s performance doesn’t go any worse, even with the extra challenge of sensor noise. This shows how strong it is.

The controller’s performance is best seen when there is both sensor noise and dynamics that are not represented. The control input is less stable than it should be, but it remains within tolerable bounds and makes the right adjustments to keep the force output near to the intended set point. The HOFA-SMC’s higher-order adjustment makes the changes in the force output less than what would be anticipated from a regular sliding mode controller. This shows that the controller can manage noise and dynamics that aren’t described, which makes it more robust than traditional control approaches.

## 8. Comparison of higher-order fully actuated sliding mode controller and feedback linearization controller

The controller is quite good at managing real-world situations because it can keep the force output steady even when there are outside factors like noise and unmodeled dynamics. The changes in the control input show that the system is making up for these problems, but the force output is steady and near to the goal value of 1.53 N. This shows that the system can handle a lot of uncertainty, which means it will work well even when things aren’t perfect.

The HOFA-SMC works well when everything is perfect, carefully following the control input and force output to the set point. The controller maintains a smooth and precise reaction, showing that it can manage the system’s changes without any outside help. The force output stays steady at around 1.53 N, the intended number. This means that the controller is working well.

The HOFA-SMC still works effectively even when sensor noise is added. The control input changes slightly, but these changes are minor, and the force output stays near the setpoint of 1.53 N. The controller does a good job of making up for the noise, which has a negligible influence on how well the system works as a whole. The controller’s performance doesn’t go any worse, even with the extra challenge of sensor noise. This shows how strong it is.

**Fig 19:**
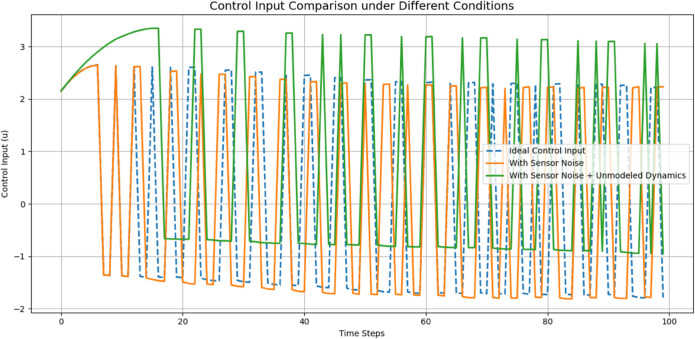
Control input comparison on different conditions. The green line represents Ideal Control Input, the orange line represents Control Input with Sensor Noise, and the blue dashed line represents Control Input with Sensor Noise and Unmodeled Dynamics.

Fig 19 shows Control Input Comparison Under Different Conditions. The controller’s performance is best seen when sensor noise and dynamics are not represented. The control input is less stable than it should be, but it remains within tolerable bounds and makes the proper adjustments to keep the force output near the intended set point. The HOFA-SMC’s higher-order adjustment makes the changes in the force output less than what would be anticipated from a regular sliding mode controller. This shows that the controller can manage noise and dynamics that aren’t described, which makes it more robust than traditional control approaches.

The controller is good at managing real-world situations because it can keep the force output steady even when outside factors like noise and unmodeled dynamics exist. With a simulated noise level of ±10% and an actuator saturation level of ±5%, the HOFA-SMC kept the highest force deviation to ±0.08 N and the joint angle inaccuracy to < 0.015 rad. This means that the system can handle noise and model uncertainty up to these levels while still being stable and converging. The changes in the control input show that the system is making up for these problems, but the force output is steady and close to the goal value of 1.53 N. This shows that the system can handle a lot of uncertainty, which means it will work well even when things aren’t perfect.

## 9. Conclusions and future work

This paper proposes non-linear control methodologies, HOFA-SMC and FLC, to compensate for the complete robotic hand model under different applied forces, torques, and unbalanced load conditions. Additionally, the outcomes of HOFA-SMC and FLC under different parametric values are explained. The complete results for the full five fingers—Index, Ring, Middle, and Little (4-DOF), and Thumb (3-DOF) are discussed. Flexion, Extension, and Grasping are performed using high-order SMC in a Python environment, and FLC is then implemented under the same test scenarios.

The results of both proposed controllers are compared with those of past research. The results show that HOFA-SMC demonstrates superiority in fast convergence, more precise trajectory, and robustness against uncertainties.

The comparison of both implemented controllers highlights apparent differences in performance across multiple parameters. HOFA-SMC is superior because it achieves 1.7% better maximum flexion angles with less improvement in flexibility. Additionally, HOFA-SMC provides better extension angles, showing a 15.8% improvement, offering the best extension. HOFA-SMC’s time of convergence is also 5 times better than FLC. Furthermore, a higher lambda value is applied, indicating robustness in the control approach.

Compared with practical versions of SMC, the implemented HOFA-SMC shows a very fast response, very high robustness, the lowest chattering, very high accuracy, and high damping, with a steady-state time of only 0.2s. For a robotic finger to replicate the characteristics of a real finger, its response time should be between 0-1s, and the results validate this approach. Both controllers successfully suppressed higher-order harmonics; however, the HOFA SMC is more reliable for practical applications due to its capacity to manage uncertainties and erratic fluctuations. Experimental validation of the full-hand model highlighted its practicality and potential for prosthetic and assistive technologies, overcoming limitations in earlier two-finger models.

This work can be implemented on a hardware platform for real-time verification in the future. Future work aims to integrate advanced control techniques such as gain scheduling and back-stepping to improve system performance further. Expanding the model to multi-fingered configurations with sensory feedback will enhance inclusivity and user experience. The scalability and adaptability of HOFA-SMC set a new benchmark for prosthetic technologies, offering precise, robust, and stable control in complex multi-joint systems.

## Supporting information

S1 Appendix Table 1. Controller and compensation parameters are essential to achieve the desired optimal system response, ensuring stability and fast convergence. They also help improve the control system to mitigate disturbances.
(DOCX)

S2 Appendix Table 2. Simulation parameters are equally important for accurate modeling and predicting behavior in real-world systems. Especially in the case of prosthetics, it is important to validate results from simulations before applying them to hardware. This reduces the risk of malfunction and provides informed values before actual implementation.
(DOCX)

S3 Appendix Table 3. Ball parameters are crucial for desired modeling. These parameters specifically affect motion and interaction with a robotic manipulator.
(DOCX)

S4 Appendix Table 4. Masses of links help us design the multi-link structure of a robotic hand. They influence system stability, force distribution, and dynamic behavior.
(DOCX)

S5 Appendix Table 5. The length of links is a fundamental parameter. All links, in contrast to finger length, are necessary. They help determine the motion range and geometry of the system. For accurate and precise control, the length is essential.
(DOCX)

S6 Appendix Table 6. Desired joint angles for flexion and extension are key parameters, especially in prosthetics and exowearable modalities. They help define the motion range to perform required tasks. By setting these angles properly, the system ensures accurate movements and functionality.
(DOCX)

S7 Appendix Table 7. Finger positions relative to the ball in 2D and 3D are essential parameters in grasping the ball or controlling a full robotic hand. The ball occupies a wide area of space, from which grasping can be easily estimated. This allows for a more comprehensive understanding of grip stability, contact forces, and force distribution.
(DOCX)

S8 Appendix Table 8. Initial angles for joints (in radians) are important for defining the starting position and orientation (pose) of the robotic hand. They help to set an accurate motion planning path to ensure the system starts operating from a controlled state.
(DOCX)

S9 Appendix Table 9. Desired joint angles for flexion and extension are key parameters, especially in prosthetics and exoskeleton modalities. They help define the motion range to perform required tasks. By setting these angles properly, the system ensures accurate movements and functionality.
(DOCX)
