# Supplementary material for: Nonlinear control of a fully actuated robotic hand using high-order sliding mode and feedback linearization controllers
Source: PLoS One. 2025 Oct 17;20(10):e0333512. doi: 10.1371/journal.pone.0333512 (PMC12533922; doi:10.1371/journal.pone.0333512)
Supplement: S1 Appendix — They also help improve the control system to mitigate disturbances. (DOCX) [file pone.0333512.s001.docx]

**S1 Appendix**

**Table 1.** Controller and Compensation Parameters of HOFA-SMC

| **Parameter** | **Value** |
| --- | --- |
| Sliding Mode Parameter (λ) | 6 |
| Proportional Gain ($k_{p}$) | 10 |
| Derivative Gain ($k_{d}$) | 7 |
| Integral Gain ($k_{i}$) | 5 |
| Switching Gain (K) | 10 |
| Saturation Boundary (δ) | 0.05 |
| Nonlinear Term Gain (λ) | 4 |
| Dynamic Compensation Factor ($\gamma_{1}$) | 0.15 |
| System Inversion Parameter (μ) | 2.2 |
