# Supplementary material for: Nonlinear control of a fully actuated robotic hand using high-order sliding mode and feedback linearization controllers
Source: PLoS One. 2025 Oct 17;20(10):e0333512. doi: 10.1371/journal.pone.0333512 (PMC12533922; doi:10.1371/journal.pone.0333512)
Supplement: S2 Appendix — Especially in the case of prosthetics, it is important to validate results from simulations before applying them to hardware. This reduces the risk of malfunction and provides informed values before actual implementation. (DOCX) [file pone.0333512.s002.docx]

**S2 Appendix**

**Table 2.** Simulation Parameters

| **Parameter** | **Value** |
| --- | --- |
| Initial Force | 0.0 N |
| Time | 5 seconds (100 samples) |
| Thumb Force | 1.53 N |
| Index Force | 1.53 N |
| Middle Force | 1.23 N |
| Ring Force | 0.92 N |
| Little Force | 0.92 N |
