# Supplementary material for: Nonlinear control of a fully actuated robotic hand using high-order sliding mode and feedback linearization controllers
Source: PLoS One. 2025 Oct 17;20(10):e0333512. doi: 10.1371/journal.pone.0333512 (PMC12533922; doi:10.1371/journal.pone.0333512)
Supplement: S3 Appendix — These parameters specifically affect motion and interaction with a robotic manipulator. (DOCX) [file pone.0333512.s003.docx]

**S3 Appendix**

**Table 3.** Ball Parameters

| **Parameter** | **Value** |
| --- | --- |
| Radius of Ball | 12.0 cm |
