# Supplementary material for: Nonlinear control of a fully actuated robotic hand using high-order sliding mode and feedback linearization controllers
Source: PLoS One. 2025 Oct 17;20(10):e0333512. doi: 10.1371/journal.pone.0333512 (PMC12533922; doi:10.1371/journal.pone.0333512)
Supplement: S4 Appendix — They influence system stability, force distribution, and dynamic behavior. (DOCX) [file pone.0333512.s004.docx]

**S4 Appendix**

**Table 4.** Masses of Links

| **Finger** | **Metacarpal (**$\boldsymbol{m}_{\boldsymbol{1}}$**)** | **Proximal (**$\boldsymbol{m}_{\boldsymbol{2}}$**)** | **Middle (**$\boldsymbol{m}_{\boldsymbol{3}}$**)** | **Distal (**$\boldsymbol{m}_{\boldsymbol{4}}$**)** |
| --- | --- | --- | --- | --- |
| Thumb (3-DOF) | 0.4 kg | 0.3 kg | 0.2 kg | N/A |
| Index (4-DOF) | 0.5 kg | 0.4 kg | 0.3 kg | 0.2 kg |
| Middle (4-DOF) | 0.5 kg | 0.4 kg | 0.3 kg | 0.2 kg |
| Ring (4-DOF) | 0.5 kg | 0.4 kg | 0.3 kg | 0.2 kg |
| Little (4-DOF) | 0.4 kg | 0.3 kg | 0.2 kg | 0.1 kg |
