# Supplementary material for: Nonlinear control of a fully actuated robotic hand using high-order sliding mode and feedback linearization controllers
Source: PLoS One. 2025 Oct 17;20(10):e0333512. doi: 10.1371/journal.pone.0333512 (PMC12533922; doi:10.1371/journal.pone.0333512)
Supplement: S5 Appendix — All links, in contrast to finger length, are necessary. They help determine the motion range and geometry of the system. For accurate and precise control, the length is essential. (DOCX) [file pone.0333512.s005.docx]

**S5 Appendix**

**Table 5.** Lengths of Links

| **Finger** | **Metacarpal (**$\boldsymbol{l}_{\boldsymbol{1}}$**)** | **Proximal (**$\boldsymbol{l}_{\boldsymbol{2}}$**)** | **Middle (**$\boldsymbol{l}_{\boldsymbol{3}}$**)** | **Distal (**$\boldsymbol{l}_{\boldsymbol{4}}$**)** |
| --- | --- | --- | --- | --- |
| Thumb (3-DOF) | 3.0 cm | 2.0 cm | 1.5 cm | N/A |
| Index (4-DOF) | 3.0 cm | 2.5 cm | 2.0 cm | 1.5 cm |
| Middle (4-DOF) | 3.2 cm | 2.6 cm | 2.1 cm | 1.5 cm |
| Ring (4-DOF) | 3.0 cm | 2.4 cm | 2.0 cm | 1.4 cm |
| Little (4-DOF) | 2.5 cm | 2.0 cm | 1.5 cm | 1.0 cm |
