# Supplementary material for: Nonlinear control of a fully actuated robotic hand using high-order sliding mode and feedback linearization controllers
Source: PLoS One. 2025 Oct 17;20(10):e0333512. doi: 10.1371/journal.pone.0333512 (PMC12533922; doi:10.1371/journal.pone.0333512)
Supplement: S6 Appendix — They help define the motion range to perform required tasks. By setting these angles properly, the system ensures accurate movements and functionality. (DOCX) [file pone.0333512.s006.docx]

**S6 Appendix**

**Table 6.** Desired Joint Angles for Flexion and Extension

| **Motion** | **Finger** | **Angles (radians)** |
| --- | --- | --- |
| Flexion | Thumb | [3.00, 2.95, 2.85] |
| Flexion | Index | [3.00, 2.95, 2.85, 2.80] |
| Extension | Thumb | [0.10, 0.12, 0.15] |
| Extension | Index | [0.10, 0.12, 0.15, 0.18] |
