# Supplementary material for: Nonlinear control of a fully actuated robotic hand using high-order sliding mode and feedback linearization controllers
Source: PLoS One. 2025 Oct 17;20(10):e0333512. doi: 10.1371/journal.pone.0333512 (PMC12533922; doi:10.1371/journal.pone.0333512)
Supplement: S7 Appendix — The ball occupies a wide area of space, from which grasping can be easily estimated. This allows for a more comprehensive understanding of grip stability, contact forces, and force distribution. (DOCX) [file pone.0333512.s007.docx]

**S7 Appendix**

**Table 7.** Finger Positions Relative to Ball (2D and 3D)

| **Finger** | **Position (x, y) 2D** | **Position (x, y, z) 3D** |
| --- | --- | --- |
| Thumb | $\left( r\cos\frac{\pi}{4},r\sin\frac{\pi}{4} \right)$ | $\left( r\cos\frac{\pi}{4}\sin\frac{\pi}{4},r\sin\frac{\pi}{4}\sin\frac{\pi}{4},r\cos\frac{\pi}{4} \right)$ |
| Index | $\left( r\cos\frac{\pi}{8},r\sin\frac{\pi}{8} \right)$ | $\left( r\cos\frac{\pi}{8}\sin\frac{\pi}{4},r\sin\frac{\pi}{8}\sin\frac{\pi}{4},r\cos\frac{\pi}{4} \right)$ |
| Middle | $\left( r,0 \right)$ | $\left( r,0,0 \right)$ |
| Ring | $\left( r\cos\left( -\frac{\pi}{8} \right),r\sin\left( -\frac{\pi}{8} \right) \right)$ | $\left( r\cos\left( -\frac{\pi}{8} \right)\sin\frac{\pi}{4},r\sin\left( -\frac{\pi}{8} \right)\sin\frac{\pi}{4},r\cos\frac{\pi}{4} \right)$ |
| Little | $\left( r\cos\left( -\frac{\pi}{4} \right),r\sin\left( -\frac{\pi}{4} \right) \right)$ | $\left( r\cos\left( -\frac{\pi}{4} \right)\sin\frac{\pi}{4},r\sin\left( -\frac{\pi}{4} \right)\sin\frac{\pi}{4},r\cos\frac{\pi}{4} \right)$ |
