# Supplementary material for: Nonlinear control of a fully actuated robotic hand using high-order sliding mode and feedback linearization controllers
Source: PLoS One. 2025 Oct 17;20(10):e0333512. doi: 10.1371/journal.pone.0333512 (PMC12533922; doi:10.1371/journal.pone.0333512)
Supplement: S8 Appendix — They help to set an accurate motion planning path to ensure the system starts operating from a controlled state. (DOCX) [file pone.0333512.s008.docx]

**S8 Appendix**

**Table 8.** Initial Angles for Joints (in radians)

| **Joint** | **Angles** |
| --- | --- |
| Thumb (CMC, MCP, IP) | [0.7854, 0.5236, 0.3927] |
| Index (MCP, PIP, DIP, Base) | [0.5236, 0.3927, 0.3142, 0.2618] |
| Middle, Ring, Little (MCP, PIP, DIP, Base) | [0.5236, 0.3927, 0.3142, 0.2618] |
