# Supplementary material for: Nonlinear control of a fully actuated robotic hand using high-order sliding mode and feedback linearization controllers
Source: PLoS One. 2025 Oct 17;20(10):e0333512. doi: 10.1371/journal.pone.0333512 (PMC12533922; doi:10.1371/journal.pone.0333512)
Supplement: S9 Appendix — They help define the motion range to perform required tasks. By setting these angles properly, the system ensures accurate movements and functionality. (DOCX) [file pone.0333512.s009.docx]

**S9 Appendix**

**Table 9.** Desired and Maximum Angles for Flexion and Extension (in radians)

| **Joint** | **Type** | **Angles** |
| --- | --- | --- |
| Thumb | Desired Flexion | [0.7800, 0.5200, 0.3900] |
| Thumb | Desired Extension | [0.7900, 0.5300, 0.4000] |
| Thumb | Max Flexion | [0.7800, 0.5200, 0.3900] |
| Thumb | Max Extension | [0.7900, 0.5300, 0.4000] |
| Index | Desired Flexion | [0.5200, 0.3900, 0.3100, 0.2600] |
| Index | Desired Extension | [0.5300, 0.4000, 0.3200, 0.2700] |
| Index | Max Flexion | [0.5200, 0.3900, 0.3100, 0.2600] |
| Index | Max Extension | [0.5300, 0.4000, 0.3200, 0.2700] |
